# Selective expansion of high functional avidity memory CD8 T cell clonotypes during hepatitis C virus reinfection and clearance

**DOI:** 10.1371/journal.ppat.1006191

**Published:** 2017-02-01

**Authors:** Mohamed S. Abdel-Hakeem, Maude Boisvert, Julie Bruneau, Hugo Soudeyns, Naglaa H. Shoukry

**Affiliations:** 1 Centre de Recherche du Centre Hospitalier de l’Université de Montréal (CRCHUM), Montréal, Québec, Canada; 2 Department of Microbiology and Immunology, Faculty of Pharmacy, Cairo University, Cairo, Egypt; 3 Département de microbiologie, infectiologie et immunologie, Université de Montréal, Montréal, Québec, Canada; 4 Département de médecine familiale et de médecine d’urgence, Université de Montréal, Montréal, Québec, Canada; 5 Centre hospitalier universitaire Sainte-Justine, Montréal, Québec, Canada; 6 Département de médecine, Université de Montréal, Montréal, Québec, Canada; The University of Chicago, UNITED STATES

## Abstract

The dynamics of the memory CD8 T cell receptor (TCR) repertoire upon virus re-exposure and factors governing the selection of TCR clonotypes conferring protective immunity in real life settings are poorly understood. Here, we examined the dynamics and functionality of the virus-specific memory CD8 TCR repertoire before, during and after hepatitis C virus (HCV) reinfection in patients who spontaneously resolved two consecutive infections (SR/SR) and patients who resolved a primary but failed to clear a subsequent infection (SR/CI). The TCR repertoire was narrower prior to reinfection in the SR/SR group as compared to the SR/CI group and became more focused upon reinfection. CD8 T cell clonotypes expanding upon re-exposure and associated with protection from viral persistence were recruited from the memory T cell pool. Individual CD8 T cell lines generated from the SR/SR group exhibited higher functional avidity and polyfunctionality as compared to cell lines from the SR/CI group. Our results suggest that protection from viral persistence upon HCV reinfection is associated with focusing of the HCV-specific CD8 memory T cell repertoire from which established cell lines showed high functional avidity. These findings are applicable to vaccination strategies aiming at shaping the protective human T cell repertoire.

## Author summary

In this study we examined the diversity and dynamics of the repertoire of receptors of CD8 T cells that are selected and enriched upon real-life multiple exposures to viral infections. Using hepatitis C virus (HCV) infection in a cohort of high risk people who inject drugs, we demonstrate that protection upon two subsequent infections was associated with a narrow repertoire of virus-specific CD8 T cells and selective expansion of cells with high polyfunctionality (increased TNFα production and cytotoxic potential). Our results have important implications in vaccination programs aiming at shaping the CD8 T cell repertoire against viral infections and cancers.

## Introduction

The capacity of CD8 T cells to recognize and respond to various pathogen-derived antigens is dictated by the diversity of their T cell receptor (TCR) repertoire. The TCR is a heterodimer of two chains, α and β, that comprise constant and variable regions. The most variable region in both chains is generated by somatic recombination involving variable (V), junction (J) and diversity (D) gene segments that could theoretically generate ~10^15–20^ unique TCRs or T-cell clonotypes capable of recognizing peptide-MHC (pMHC) complexes [1]. Positive and negative selection in the thymus leaves ~ 2x10^7^ T-cell clonotypes with unique TCR amino acid sequences that constitute the naïve human T-cell repertoire. Hypervariable complementarity-determining regions 1 and 2 (CDR1 and CDR2) are formed by the germline V region sequences and interact mainly with MHC. The CDR3 of the TCR α and β chains, the most variable region of the TCR, are encoded by the V(D)J junction and interact primarily with peptide, thus determining antigenic specificity of the TCR [1]. Upon exposure to a viral infection, particular clonotypes recognizing virus-derived epitopes/pMHC are selected and expand into primary effectors that then contract to form a pool of long-lived memory T cells that are able to respond rapidly upon virus re-exposure. The size and diversity of the expanding effector T cell repertoire can vary according to the initial germline repertoire of naïve CD8 T cells and strength of interaction with pMHC (affinity and avidity). In contrast, factors governing the size and diversity of the antiviral memory CD8 T cell pool are not well understood. Most importantly, determinants for selection and maintenance of CD8 T cell clonotypes exerting an efficacious and protective anti-viral immune response upon virus re-exposure or reactivation remain elusive. There is evidence to suggest that the memory CD8 T cell repertoire can be modulated by heterologous infections and age [2, 3] but other host and viral factors could be involved.

One key characteristic of the virus-specific TCR repertoire is diversity, which defines the number of unique clonotypes forming the repertoire. The repertoire can be characterized as «narrow» or «broad» depending on the number of unique clonotypes it contains. Distinctive molecular properties of T cell clonotypes that determine their functionality include affinity, avidity, functional avidity and flexibility. Affinity describes the strength of binding of a single TCR to cognate pMCH complexes, whereas avidity (structural avidity) is the sum of binding affinities of multiple TCRs to their pMHC complexes. Functional avidity depends on how this translates into measurable biological functions such as cytokine production [4]. Flexibility is the capacity to recognize multiple variants of the same epitope and cross-react with these variants. Although TCRs are generally very specific or «private» in their response to a pMHC complex, identical TCR sequence usage in response to a specific epitope across multiple individuals termed «public TCRs» were observed in a number of infections, tumors and even autoimmune conditions [5, 6].

Studies in chronic cytomegalovirus (CMV) and Epstein-Barr virus (EBV) infections demonstrated focusing and increased affinities of the virus-specific CD8 TCR repertoire [7]. In human immunodeficiency virus (HIV) infection specific clonotypes dominated the response against an epitope in the p24 Gag (KK10; residues 263–272) restricted by HLA B*2705 in patients who controlled viral replication. These clonotypes exhibited higher avidity and polyfunctionality and superior control of HIV replication *in vitro* [8–10]. Furthermore, certain public clonotypes were detected in several HIV controllers [11, 12]. However, another study examining different epitopes did not observe preferential use of particular clonotypes [13]. Escape mutations in targeted epitopes could be recognized by some of these highly functional clonotypes [10] yet they also drove the expansion of alternate clonotypes with dual reactivity against both the original and mutated epitopes [14]. This expansion was inversely correlated with residual viral load, suggesting that these alternative clonotypes play a role in limiting replication of mutated viruses [15].

Hepatitis C virus (HCV) infection represents a unique model of a human viral infection with dichotomous outcomes, i.e. spontaneous clearance (~30% of infected individuals) and persistent infection [16]. Control of primary HCV infection in the chimpanzee model was associated with a more diverse CD8 TCR repertoire than infections that became chronic and were associated with escape mutations [17]. In humans, selection of high-avidity CD8 T cells correlated with control of primary infection [18]. Analysis in individuals with long-term clearance or persistent infection demonstrated a biased TCR repertoire towards a common core of public TCRs irrespective of infectious outcome [19]. HCV is also thought to exploit a «hole» in the T cell repertoire to undergo escape mutation and evade detection by the immune system [20]. The dynamics of the repertoire upon re-exposure to HCV are less defined. Rechallenging chimpanzees following clearance of primary infection demonstrated that resolution temporally coincided with the expansion of dominant clonotypes that were associated with clearance of primary infection [21]. This is consistent with data in the lymphocytic choriomeningitis virus (LCMV) model [22]. Higher CDR3 diversity correlated with viral clearance and better control of escape mutations within the targeted epitope and temporary narrowing of the repertoire was observed at the peak of the recall response [17].

The majority of acute HCV infections in North America occur among people who inject drugs (PWID). Individuals who spontaneously clear their primary infection but maintain risk behaviors associated with HCV re-exposure remain at high risk of reinfection. As such, HCV represents a unique model to study correlates of protective immunity in a real life challenge experiment. Follow-up of PWIDs through consecutive episodes of infection and reinfection can provide an insight into the dynamics of the virus-specific TCR repertoire and the functional properties associated with protective immunity. We have previously demonstrated that protection from chronicity upon reinfection with HCV correlated with expansion of polyfunctional HCV-specific effector T cells and increased breadth of T cell responses, suggesting the generation of *de novo* responses. In contrast, viral persistence was associated with limited expansion of virus-specific T cells and infection with variant viral strains that were not recognized by preexisting virus-specific memory T cells [23].

Here, we examined dynamics of the TCR repertoire during reinfection. Our objectives were to distinguish the role of pre-existing memory versus *de novo* T cell responses during reinfection and to establish the functional correlates of CD8 T cell clonotypes associated with protective immunity in real life exposure and reinfection. Our results suggest that dominant HCV-specific tetramer+ CD8 T cell clonotypes mobilized during the reinfection episode were exclusively recruited from the preexisting memory pool. Protection from chronic infection was associated with a narrower TCR repertoire that became more focused upon reinfection with preferential selection of TCR clonotypes with high functional avidity and polyfunctionality.

## Results

### HCV-specific dominant and sub-dominant clonotypes at peak reinfection are recruited from the preexisting memory population

To examine the evolution and dynamics of the HCV-specific memory CD8 T cell repertoire upon virus re-exposure, we performed longitudinal analysis of this repertoire on virus-specific CD8 T cells during HCV reinfection episode in five patients with different outcomes (S1 Table). Three patients resolved two successive HCV infections, hereinafter termed SR/SR. Two patients failed to spontaneously resolve the reinfection episode and developed chronic viremia despite clearing a previous HCV infection, hereinafter termed SR/CI.

**Fig 1:**
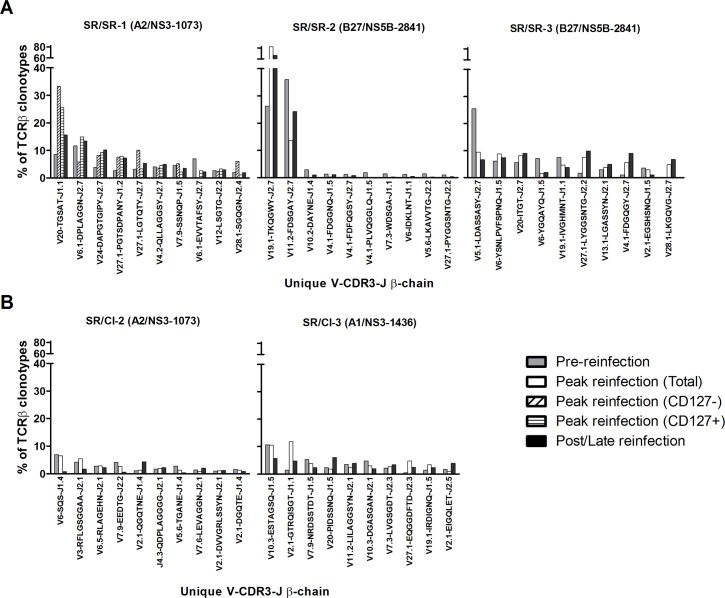
HCV-specific tetramer+ CD8 T-cell clonotypes mobilized during the reinfection episode were exclusively recruited from the pre-existing memory population. The top ten dominant clonotypes (frequency ≥1%) isolated directly ex vivo from (A) three SR/SR patients and (B) two SR/CI patients followed-up longitudinally during reinfection episode at pre-reinfection, peak expansion and post/late reinfection (all five patients). For patient SR/SR-1, at the peak of reinfection, cells were sorted according to CD127 expression into CD127- population (effector population, diagonally stripped white bar) and CD127+ (memory population, horizontally stripped white bar). Tetramers used in each patient are indicated between brackets next to the patient number.

Epitope-specific CD8+ T cells identified by three different MHC class I tetramers were sorted and the TCR repertoire sequenced at three distinct time points for each patient: i) pre-reinfection (range: -55 to -20 weeks before detection of reinfection), ii) peak reinfection (range: 3 to 8 weeks post detection of reinfection), and iii) late/post reinfection follow-up (range: 12 to 24 weeks post detection of reinfection). The peak reinfection time point was selected based on our previously published longitudinal analysis of the frequency of tetramer+ CD8 T cells [23]. In addition, we examined the repertoire during primary acute infection in patient SR/SR-3, for whom samples were available early at week 3 post primary infection. In patients SR/SR-1 and SR/SR-3, tetramer frequency was high enough during peak reinfection and primary infection, respectively which allowed us to sort effector (CD127-) and memory (CD127+) HCV-specific CD8+ T cells as well. As a control, total naïve CD8+ T cells from patients SR/SR-1 and SR/CI-2 defined as CD8+CD45RO- were sorted and sequenced for all three time points tested. A summary of the time points tested for each patient and the corresponding tetramer frequencies is presented in S1 Table. The gating strategy and post-sorting purity are presented in S1 and S2 Figs. A summary of the sequencing information including the number of cells, sequences and clonality is presented in S2 Table. TCRBV and TCRBJ gene usage, as well as the CDR3 amino acid sequence of all TCR β chains expressed by the different HCV-specific T cell clonotypes were analyzed (S3–S7 Tables). The top 10 clonotypes for each patient and time point are presented in Fig 1. Longitudinal analysis of the TCR β chain dynamics demonstrated a striking difference between SR/SR and SR/CI patients where there was high level expansion (up to 3.5 fold) of specific clonotypes in two out of three SR/SR patients upon reinfection but limited or no expansion in patient SR/SR-3 and both SR/CI patients. This is consistent with the limited expansion of tetramer+ cells observed in the SR/CI (S1 Table and [23]). The analysis also demonstrated that the dominant (frequency > 1%) and sub-dominant (frequency > 0.5%) clonotypes at the peak reinfection time point were recruited from the pre-existing memory CD8 T cell populations and no new clonotypes were detected. This was also true in patient SR/SR-1 where we managed to examine the repertoire of effector (CD127-) CD8 T cells that could be indicative of a *de novo* T cell response. Comparison of the effector (CD127-) and memory (CD127+) CD8 T cell repertoire showed the same clonotypes albeit at slightly different frequencies (Fig 1 and S3–S7 Tables). Although, some new clonotypes that were not present at pre-reinfection were detected in all patients at the peak time point as shown in the Venn diagrams in S3 Fig, they were of low frequencies (< 0.5%). Similarly, comparison of the repertoire during primary acute infection and reinfection in patient SR/SR-3 demonstrated that the repertoire generated during primary infection was relatively stable and a precursor to the memory repertoire (S4 Fig).

Although the clonotype composition of the T cell repertoire remained relatively unchanged upon reinfection, there was a change in the dominance and hierarchy of the different clonotypes where a more limited number of clonotypes dominated the response at the peak of reinfection. For example, patient SR/SR-1 exhibited preferential expansion of the V20 clonotype. This is even more evident in patient SR/SR-2 where the two most dominant clonotypes (V19.1 and V11.2) switched hierarchies upon reinfection, suggesting a selection and amplification of particular clonotypes during reinfection.

Our analysis demonstrated no overlap and no common clonotypes within the dominant and sub-dominant Vβ-CDR3-Jβ detected in the tested patients. This lack of common repertoire was observed among patients targeting the same epitope whether they belonged to different groups (SR/SR-1 and SR/CI-2 recognizing the HLA-A2 restricted NS3-1073 epitope) (Fig 1 and S3 and S6 Tables) or the same group (SR/SR-2 and SR/SR-3 recognizing the HLA-B27 restricted NS5B-2841 epitope) (S4 and S5 Tables).

Altogether, these results suggest that the dominant protective CD8 T cell clonotypes expanding at the peak of reinfection episode were recruited from the preexisting memory pool and at least within this set of individuals, no common or public TCRs were detectable. Very limited expansion was observed in HCV-specific CD8 T cells and specific clonotypes in the SR/CI group.

### Protection against viral persistence upon HCV reinfection is associated with focusing of the virus-specific T cell repertoire

**Fig 2:**
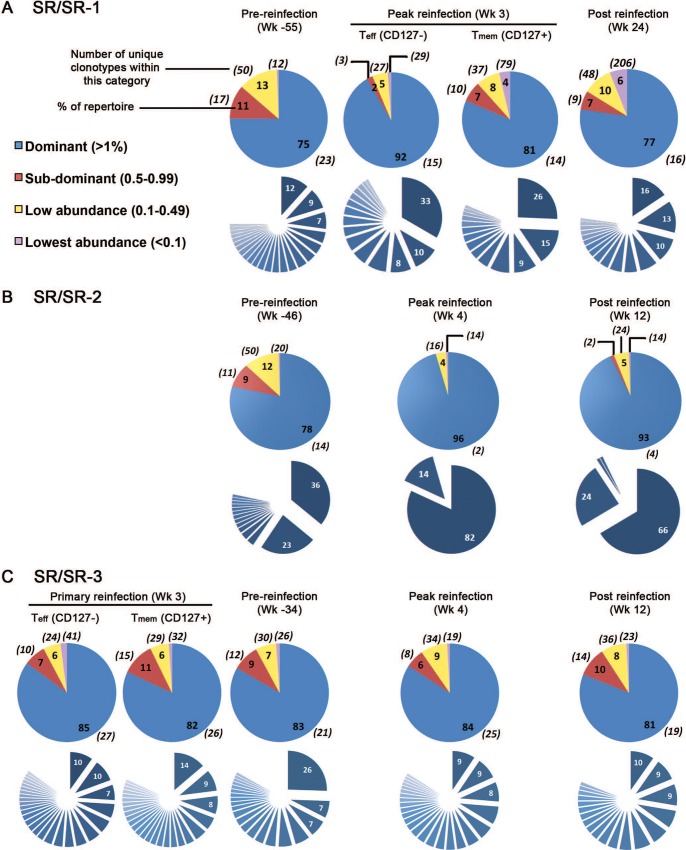
Narrowly focused epitope-specific CD8 T cell repertoire in SR/SR patients. Distribution of the different categories (dominant, sub-dominant, low-abundance and lowest abundance clonotypes in blue, red, yellow and violet, respectively) with respect to the total clonotypes forming the epitope-specific CD8 T cell repertoire for three SR/SR patients at pre-reinfection, peak and post reinfection episode (A) patient SR/SR-1 (B) SR/SR-2 and (C) SR/SR-3. Patient SR/SR-3 was followed-up during primary HCV infection, as well. The pie charts in the upper rows show the percentage of each category with respect to the total repertoire. The percentages are represented by numbers inside the pie. The numbers between brackets around the pie charts represent the number of unique clonotypes forming each category. The sliced pie charts in blue in the lower rows represent the dissection of the individual clonotypes forming the dominant category, with the frequency of the three most dominant clonotypes indicated in white inside the corresponding slice.

**Fig 3:**
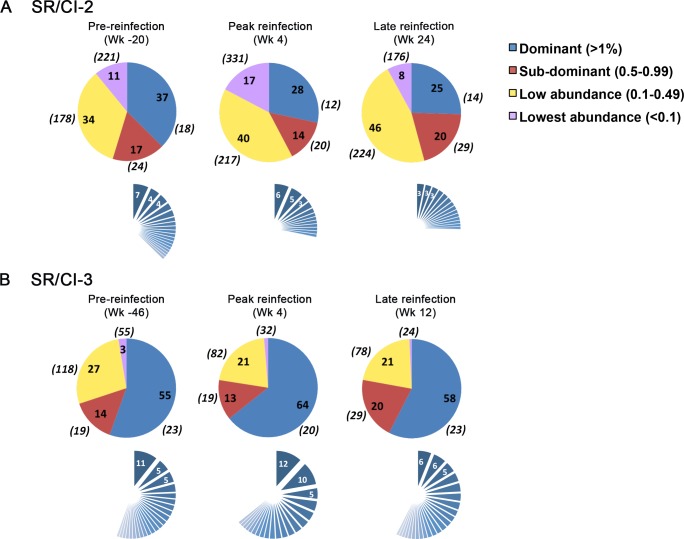
Highly diverse epitope-specific CD8 T cell repertoire in SR/CI patients. Pie charts showing the distribution of the different categories of clonotypes forming the epitope-specific CD8 T cell repertoire at pre-, peak and late during the reinfection episode in patient SR/CI-2 (A) and SR/CI-3 (B). The pie charts in the upper rows show the percentage of each category (dominant, sub-dominant, low-abundance and lowest abundance clonotypes in blue, red, yellow and violet, respectively) with respect to the total clonotypes. The percentages of each category are represented by numbers inside the pie. The numbers between brackets around the pie charts represent the number of unique clonotypes forming each category. The sliced pie charts in blue in the lower rows represent the dissection of the individual clonotypes forming the dominant category, with the frequency of the three most dominant clonotypes indicated in white inside the corresponding slice.

Data from the chimpanzee model suggested that a more diverse repertoire, i.e. the presence of a larger number of unique clonotypes recognizing the same pMHC, was associated with spontaneous resolution of primary acute HCV [17]. Hence, we proceeded to examine repertoire diversity within our study subjects in a memory response. Clonotypes were classified into 4 categories according to their frequencies within the repertoire: (i) dominant clonotypes (frequencies >1%); (ii) sub-dominant clonotypes (0.5–0.99%); (iii) low abundance clonotypes (0.1–0.49%); and (iv) lowest abundance clonotypes (<0.1%). Our analysis demonstrated that the clonotypic profile in SR/SR patients was less diverse than that observed in SR/CI patients at the pre-reinfection time point (Figs 2 and 3). In the SR/SR group, 75–83% of the repertoire was contributed by 14–23 TCR clonotypes. This repertoire became more focused at peak reinfection for patients SR/SR-1 and SR/SR-2, where 92% of the repertoire of tetramer+ CD8+ CD127- cells in SR/SR-1 and 96% of the repertoire of tetramer+ CD8+ T cells in patient SR/SR-2 were contributed by 15 and 2 clonotypes, respectively (Fig 2A and 2B). In these two patients, the repertoire retained a more focused status post reinfection when compared to the pre-reinfection time point, with 77% and 93% of the repertoire contributed by 16 and 4 clonotypes, respectively (Fig 2A and 2B). Patient SR/SR-3, for whom samples were available during primary infection, also demonstrated a focused repertoire of effector (CD127-) and memory (CD127+) HCV-specific T cells early during acute primary infection where 85% and 82% of the repertoire were represented by 27 and 26 clonotypes, respectively (Fig 2C). This repertoire remained more or less stable during the memory phase where 83% of the repertoire was represented by 21 clonotypes but did not sensibly change upon reinfection. This is consistent with our previous results, where no significant expansion of the tetramer+ population was observed in this patient upon reinfection (S1 Table), and IFNγ Enzyme-Linked Immunospot (ELISPOT) assays suggested that he had been reinfected with a different HCV subtype [23].

In contrast, the repertoire in the two SR/CI (unprotected) patients was more diverse at the preinfection time point. Dominant clonotypes represented 37% and 55% of the repertoire (18 and 23 TCR clonotypes, respectively). At the peak of the immune response during reinfection, the repertoire remained more diverse than in the SR/SR patients (the dominant category represented 28–64% of the repertoire) (Fig 3A and 3B). The remaining 40–60% of the repertoire was represented by at least 133 and as much as 568 clonotypes. In contrast, the number of minor clonotypes in the SR/SR patients was much lower (Fig 2). Furthermore, we did not observe increased focusing of the repertoire in SR/CI patients at the late reinfection time point.

The presence of a less diverse repertoire that becomes more focused upon reinfection in the SR/SR group was further supported by the observation that the top three clonotypes in patients SR/SR-1 and the top two clonotypes in patient SR/SR-2 represented 28% and 59% of the repertoire pre-reinfection, respectively (Fig 2A and 2B, dissected pie-charts in the lower rows). Upon reinfection, these clonotypes became more dominant, representing 51% and 96% of the repertoire, respectively. This increased dominance was maintained post-reinfection, where they formed 39% and 90% of the repertoire, respectively. On the other hand, the top three clonotypes in SR/CI patients never represented more than 27% of the repertoire, and were sometimes as low as 9% (Fig 3A and 3B, dissected pie-charts in the lower rows). These results confirm that the SR/SR TCR repertoire is highly focused and becomes more focused upon re-exposure and reinfection.

### Changes in diversity, richness and evenness of the repertoire during reinfection

**Fig 4:**
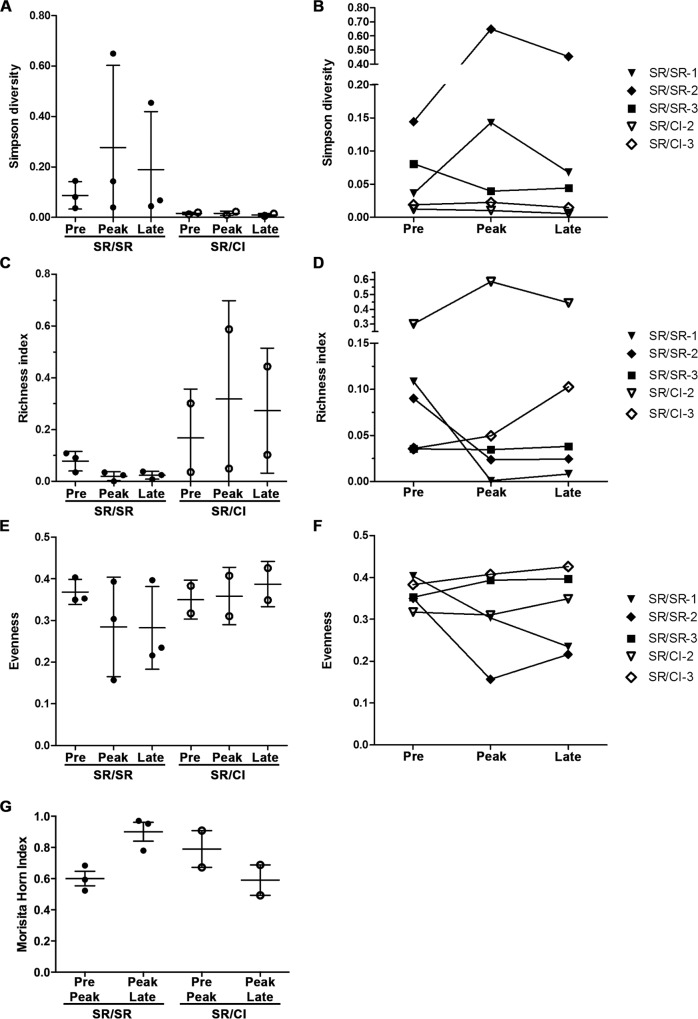
Decreased diversity, richness and evenness during reinfection in SR/SR patients. Simpson Diversity of the repertoires shown for all samples at the different time points (A) or longitudinally stratified by patient (B). Calculated as described by Simpson [24], with 0 defined as infinite diversity and 1 as no diversity. Richness index of the repertoires shown for all samples at the different time points (C) or longitudinally stratified by patient (D) represent the number of unique sequences as a ratio of the number of input cells. Evenness of the repertoires shown for all samples at the different time points (E) or longitudinally stratified by patient (F), representing the relative abundance of each clonotypes forming one repertoire. (G) Morisita Horn index calculated with the ImmunoSeq 3.0 tool to compare pre-reinfection with peak reinfection time points as well as peak reinfection with late reinfection time points.

In order to establish a quantitative measure of the changes observed in the TCR repertoire during reinfection we examined diversity using the Simpson diversity index that takes into account the number of species present as well as the abundance of each species, with 0 defined as infinite diversity and 1 as no diversity [24, 25]. The focusing of the repertoire in the SR/SR group was reflected by an increase in the Simpson diversity index for patients SR/SR-1 and SR/SR-2 indicating a less diverse repertoire but it remained stable or decreased in patient SR/SR-3 and in the SR/CI group where no expansion was observed (Fig 4A and 4B). Next, we examined richness of the repertoire. This parameter measures the number of clonotypes per sample [26]. We observed decreased richness index in the SR/SR group (mostly SR/SR-1 and SR/SR-2) indicating focusing of the repertoire while the same index remained stable in patient SR/SR-3 and slightly increased in the SR/CI group (Fig 4C and 4D). Next, we examined evenness of the repertoire. This parameter measures the relative abundance of the different clonotypes [26]. Again, we observed decreased evenness at peak reinfection in patients SR/SR-1 and SR/SR-2 indicating focusing of the repertoire (Fig 4E and 4F) while the same index slightly increased in patient SR/SR-3 and in the SR/CI group. Finally, we used the Morisita Horn index to compare the repertoires from pre-reinfection and peak reinfection, as well as from peak reinfection and late reinfection. This index is indicative of the overlap between two samples as it takes into account both the number of clonotypes and their frequencies within the repertoire [27]. An index of 1 represents complete overlap or identical repertoire and an index close to 0 represents two very different repertoires with almost no clonotypes shared between the samples. The analysis for SR/SR patients showed a Morisita Horn index around 0.6 when comparing the pre-reinfection and peak reinfection time points, indicating changes in the repertoire between these two time points. The repertoire was stable between peak reinfection and late reinfection as indicated by a Morisita Horn index around 0.9. We observed the opposite trend for SR/CI patients, where the repertoire underwent more changes between the peak reinfection and late reinfection (Morisita Horn index of 0.6) time points as compared to the changes between pre-reinfection and peak reinfection (Morisita Horn index of 0.8) suggesting increased diversification with establishment of chronic infection. Collectively, all four measures demonstrate focusing of the repertoire in the SR/SR group and unchanged or even slight increase in diversity in the SR/CI group.

### Altered CDR3 length distribution in the protected SR/SR group

**Fig 5:**
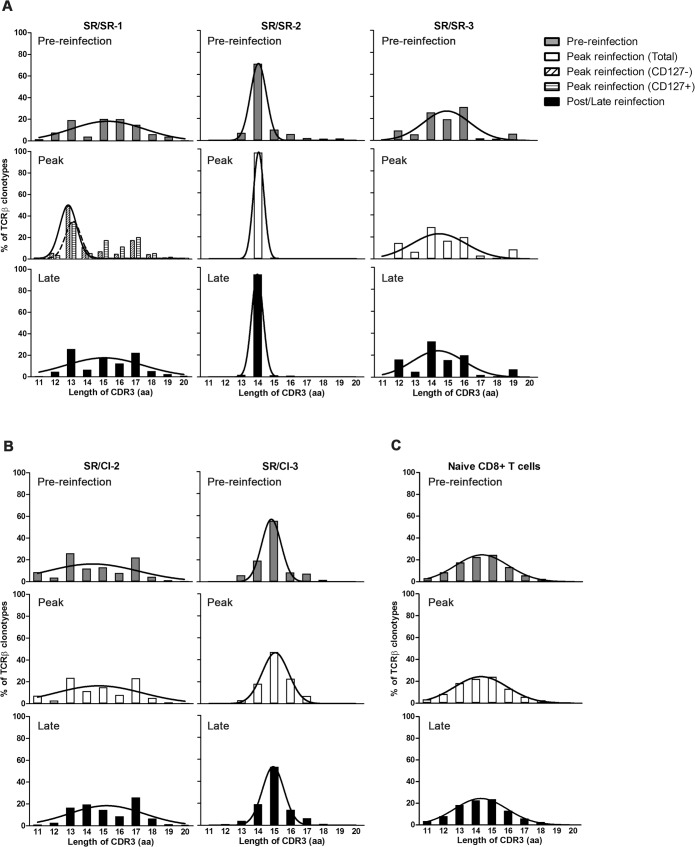
CDR3-length distribution for epitope-specific CD8 T cell repertoire in SR/SR patients as compared to SR/CI patients. Distribution of the CDR3 amino acid (aa) lengths frequencies for the clonotypes forming the epitope-specific CD8 T cell repertoire for (A) three SR/SR patients and (B) two SR/CI patients pre-, at the peak and post/late the reinfection episode. Non-linear Gaussian regression curves were added to all graph to appreciate the deviation from the normal distribution. For patient SR/SR-1, at peak reinfection time point, the plain regression line represents the CD127- population and the dotted line represents the CD127+ population. As a reference, the normal bell shaped curve from one representative naive sample (SR/CI-2) displaying a normal distribution of CDR3 length is shown in (C).

Next, we examined CDR3 amino acid (aa) length distribution. As a reference of CDR3 lengths distribution from an unselected repertoire, we analyzed TCR sequences of naive CD8 T cells sorted from patient SR/CI-2 (Fig 5C). As shown by the bell-shaped curve, the naive repertoire displayed a normal Gaussian distribution. The distribution was identical in the naive compartment in the three time points tested (preinfection, peak and follow-up). In contrast, this normal distribution was altered in HCV-specific T cells from all patients analyzed (Fig 5A and 5B), reflecting the antigen-specific selected population. Furthermore, this analysis showed an expected bias towards the CDR3 lengths of the most dominant clonotypes. For example, the CDR3 length distribution for patient SR/SR-2 was highly biased towards a length of 14 aa, which reflects the fact that the 2 highly-dominant clonotypes (representing 78–96% of the repertoire at the different time points) possess CDR3 of that length (Fig 5A, middle). In addition, for patient SR/SR-1 the CDR3 length distribution was biased towards CDR3s with a length of 13 aa (clonotype TCRVB20-TCRVJ01.01, with frequencies of 8–35% of the repertoire, Fig 5A, left). Another important observation from this analysis is that, in the SR/SR group, we could clearly see an increase in the dominance of CDR3 of certain length between the pre-reinfection time point and the peak reinfection time point (13 aa in SR/SR-1 and 14 aa in SR/SR-2). This is in agreement with the focusing of the repertoire in those patients. In the SR/CI group (Fig 5B), we also observed a bias towards the length of the most abundant clonotypes (13 and 17 aa for SR/CI-2 and 15 aa for SR/CI-3, Fig 5B) but the CDR3 length distribution remained similar across the different time points. This reflected our earlier observation of limited to no focusing of the repertoire during reinfection in these patients. Next, we compared average CDR3 lengths, nucleotides (NT) addition and Germline index measuring junctional diversity for all patients as described by Yu et al [28], but there was no clear difference between groups or across time points (S5 Fig) further confirming our earlier observation of limited novel diversification in the repertoire upon reinfection.

### Comparable TCR avidity toward MHC-peptide for individual cell lines from SR/SR and SR/CI patients

**Fig 6:**
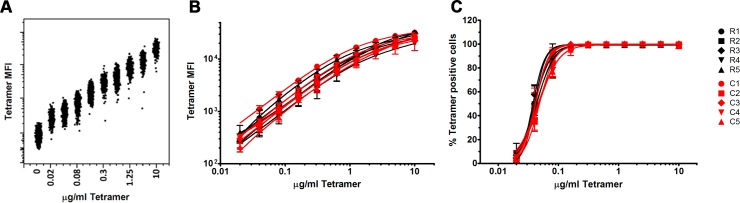
Comparable TCR avidity for individual T cell lines isolated form SR/SR and SR/CI patients. T cell lines were generated from patients SR/SR-1 (Cell lines R1 to R5) and SR/CI-2 (Cell lines C1 to C5) as described in Materials and Methods were stained with A2/NS3-1073 tetramer at a range of concentrations (0.02–10μg/ml, two fold dilutions). Data are expressed as mean +/- SD of duplicate samples in two independent experiments. (A) Representative FACS plot of single cell tetramer fluorescence intensity (MFI). (B-C) Tetramer titration curves. Mean fluorescence intensity (MFI, (B)) for each cell line/concentration and percentage (C) of tetramer positive cells for each cell line/concentration.

Studies in HIV infection have demonstrated that TCR avidity towards pMHC correlated with CD8 T cell functionality, better control of viral replication and overall lower viral loads [8, 29, 30]. So we sought to evaluate whether selection and expansion of specific clonotypes during HCV reinfection was associated with higher affinity or functional avidity that would endow them with a superior protective capacity. To establish individual cell lines specific to the HLA A2 restricted NS3 epitope (A2/NS3-1073; CINGVCWTV), HCV tetramer+CD8+ T cell were sorted and cultured under limiting dilution. Cell lines were generated from two patients: SR/SR-1 (69 cell lines generated) and SR/CI-2 (36 cell lines generated). Five cell lines from each patient were selected for detailed analysis termed hereinafter cell lines R1-R5 and cell lines C1-C5 generated from patients SR/SR-1 and SR/CI-2, respectively. We first evaluated the avidity of individual cell lines, a parameter that measures both binding strength and the total number of interactions on the T cell surface using a tetramer dilution assay. Both the percentage of tetramer positive cells and the mean fluorescence intensity (MFI) were equivalent for all cell lines regardless if they originated from a SR/SR or a SR/CI patient (Fig 6).

### Higher functional avidity and polyfunctionality for cell lines from SR/SR compared to cell lines from SR/CI

**Fig 7:**
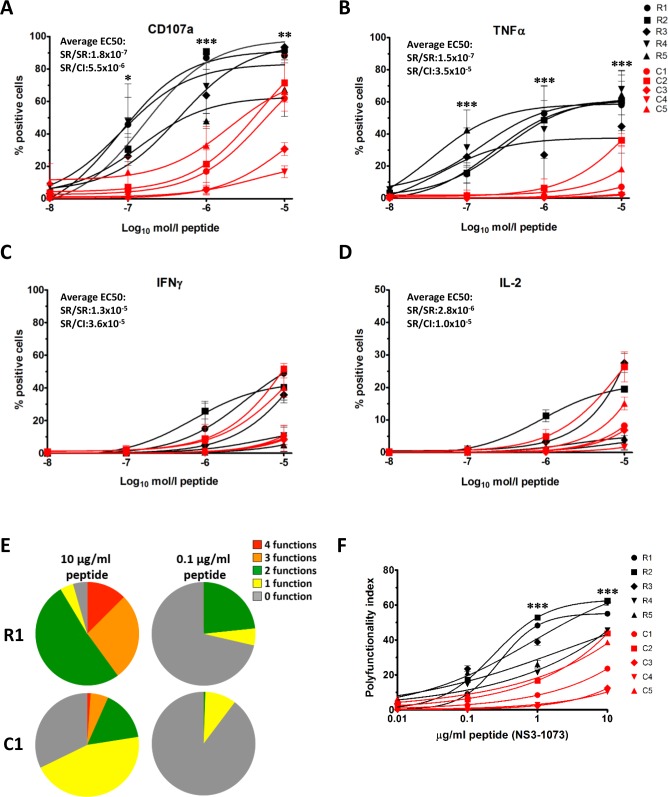
Higher functional avidity and polyfunctionality for cell lines from resolvers SR/SR compared to cell lines from chronic SR/CI. CD8 T cell lines were stimulated with autologous BLCLs prepulsed with increasing concentrations of the cognate peptide (NS3-1073) for 6h. Surface and intracellular staining was then performed as described in Materials and Methods to examine functionality by flow cytometry. Boolean gating and analysis using SPICE software was used to assess polyfunctionality profile for each clone established from patient SR/SR-1 (Cell lines R1 to R5) or from patient SR/CI-2 (Cell lines C1 to C5). (A-D) Functionality curves for (A) CD107a, (B) TNFα, (C) IFNγ and (D) IL-2. Each point represents the percentage of positive cells following stimulation with the indicated peptide concentration. Data are expressed as mean +/- SD of duplicate samples in two independent experiments. EC50 represent the concentration of peptide needed to reach half maximal production and is shown as the average of all cell lines tested for either resolver SR/SR or chronic SR/CI cell lines. (E) Representative polyfunctionality pie charts representing the percentage of cells with no function (grey); 1 function (yellow); 2 functions (green); 3 functions (orange) and 4 functions (red). (F) Polyfunctionality index of one representative experiment in duplicate representing the degree of polyfunctionality of each cell line at each peptide concentration. Calculated as described in [31]. Statistical differences between cell lines from SR/SR and SR/CI patients were calculated by two-way Anova (* p<0.05; ** p<0.01; *** p<0.001)

Next, we evaluated the functionality of individual cell lines or micropopulations in response to stimulation with the cognate NS3-1073 peptide in an intracellular cytokine staining (ICS) assay. We evaluated the surface expression of the degranulation marker CD107a and intracellular expression of TNFα, IFNγ and IL-2 (Fig 7A–7D). Representative intracellular cytokine staining (ICS) data are presented in S6 Fig. As shown in Fig 7A and 7B, CD8 T cell lines generated from the SR/SR patient were more sensitive to lower peptide concentrations and showed enhanced CD107a expression (average EC50 SR/SR = 1.8x10^-7^; SR/CI:5.5x10^-6^) and TNFα production (average EC50 SR/SR = 1.5x10^-7^; SR/CI:3.5x10^-5^). The capacity to produce IFNγ and IL-2 was clone dependent, and no clear difference was observed between cell lines from the SR/SR versus cell lines from the SR/CI patient (Fig 7C and 7D). In general, production of IL-2 was low (Fig 7D).

The polyfunctionality of CD8 T cells measured as the capacity of single cells to produce multiple functions is a key determinant of spontaneous resolution of HCV infection [32, 33]. Boolean gating and pie chart analysis demonstrated that cell lines generated from the SR/SR-1 patient had a higher degree of polyfunctionality compared to cell lines generated from the SR/CI-2 patient (Fig 7E and Supplementary S6B and S6C Fig). At maximum peptide concentration, we observed that an average of 85% of the cells displayed at least two functions for cell lines generated from SR/SR-1, compared to 30% of the cells for cell lines generated from SR/CI-2 (Fig 7E and S6B and S6C Fig). Furthermore, at limited peptide concentration (0.01μg/ml), an average of 50% of the cells were positive for at least one function for SR/SR-1 cell lines compared to an average of 13% for SR/CI-2 cell lines. Polyfunctionality index was calculated as previously described [31] to compare the overall polyfunctionality of all cell lines. As shown in Fig 7F, the degree of polyfunctionality was higher for all SR/SR-1 cell lines compared to SR/CI-2 cell lines. These results suggest that secondary clearance upon reinfection is associated with the presence of polyfunctional CD8 T cell clonotypes.

### Correlation between functionality and prevalence of specific clonotypes

To determine whether the functionality of the individual cell lines reflected preferential expansion *in vivo* during reinfection, we have sequenced the TCR of all 10 cell lines tested from patient SR/SR-1 (cell lines R1-R5) and SR/CI-2 (cell lines C1-C5). As demonstrated in S8 Table, all cell lines isolated from patient SR/SR-1 with the exception of clone R3 carried TCR clonotypes that were detectable at frequencies ranging from 0.7% to 33% at the peak of the immune response during reinfection although some of them were micropopulations composed of two different clonotypes. Nevertheless, cell line R1, one of the best responding cell lines, was partially (7%) composed of TRBV-20 that showed 33% expansion during peak reinfection. Similarly, cell line R5, with the highest TNF-α production was partially (12.5%) composed of TRBV27-01*01 that showed 8% expansion during peak reinfection. Altogether, these data demonstrate that secondary clearance upon HCV re-exposure and reinfection is associated with selective expansion of high functional avidity CD8 T cell clonotypes.

## Discussion

Defining the correlates of protective immunity at the clonotypic level is essential for fine-tuning the design of prophylactic vaccines against chronic viruses with highly variable sequences such as HIV and HCV. This study provides an insight into the dynamics of the virus-specific CD8 T cell repertoire during HCV re-exposure and reinfection in a real-life setting. Our results demonstrate that protective immunity and rapid virus clearance upon reinfection was associated with expansion of a limited number of polyfunctional CD8 T cell clonotypes selected from the memory pool. These CD8 T cells displayed a focused repertoire and cell lines established from one SR/SR patient were characterized by high functional avidity and polyfunctionality. In contrast, very little expansion of HCV specific CD8 T cell was observed in SR/CI patients who developed persistent viremia upon reinfection and their repertoire was more diverse. Cell lines established from one SR/CI patient displayed reduced functional avidity demonstrated by a much weaker production of cytokines and lower cytotoxic potential that could potentially have facilitated virus persistence and chronicity. Our observations with the cell lines reflected the *ex vivo* characterization of the functionality of HCV-specific CD8 T cells in these patients [23].

Our longitudinal analysis of the TCR repertoire, using three different HCV tetramers, showed conserved clonotype usage within the same individual where the dominant and sub-dominant clonotypes forming the effector population at the peak of the immune response during reinfection were recruited from the pre-existing memory T cell pool generated following clearance of the primary infection. New clonotypes were only detected at low abundance frequencies. Two out of three patients in the SR/SR group had dominant clonotypes following primary infection and the same clonotypes expanded upon reinfection with a homologous variant, suggesting that these particular clonotypes played an important role in viral clearance. The third SR/SR patient displayed a focused repertoire before reinfection but showed limited expansion during reinfection. We have previously shown that this patient was reinfected with a different HCV subtype (genotype 1b after primary infection with genotype 1a) which could affect his immune response. In contrast, there was very limited expansion of HCV specific CD8 T cells in the SR/CI group, even despite reinfection with the same HCV subtype for patient SR/CI-2 (Fig 1). It is noteworthy that our previous analysis of autologous virus sequences demonstrated that the SR/CI patients were infected with variant viruses that were not recognized by pre-existing memory T cells [23]. It was not possible to sequence virus from the primary infection in those patients, preventing a clear comparison between both infection episodes. That potential mismatch between the two infecting viral strains could be responsible for the lack of significant expansion of the pre-existing memory T cells or the generation of new clonotypes. Our results are in concordance with data from the LCMV model demonstrating that the TCR repertoire of the primary epitope-specific CD8 T cell response was conserved in the memory pool, and that after a secondary effector recall response, there was 60–100% identity between the clonotypes of the primary effector, memory and recall responses [22, 34]. Rapid resolution of HCV infection upon rechallenge in chimpanzees also coincided with the expansion of T cell clonotypes that dominated the memory CD8 T cell pool [17, 21].

Cross reactivity between NS3-1073–specific CD8 T cells and an influenza virus epitope (NA-231) was previously reported [35, 36]. In addition, CD8 T cells reactive to this epitope could be amplified from a significant proportion of healthy individuals with no prior exposure to HCV [35, 36]. Thus, it is possible that this may have influenced the specific CD8 T cell repertoire analyzed in our study. However, in these previous studies, the overall HCV-specific immune response was narrowly focused towards the NS3 region, which is not the case in patients SR/SR-1 and SR/CI-2 as we have previously described using ELISPOT assays [23]. Furthermore, it was previously demonstrated that these influenza cross-reactive T cells were of low affinity/avidity, and are unlikely to play a major role against HCV infection [37]. Indeed, in our hands, individual T cell lines generated from either the SR/SR or the SR/CI patient exhibited comparable avidities. Finally, TCR repertoire of cross reactive CD8 T cells was previously shown to be “private” as it varied greatly from one patient to another, suggesting that these cells do not carry a specific dominant receptor [38]. Future analysis using a larger cohort of patients responding to this epitope will be required to clarify this point.

The TCR repertoire was narrower and less diverse in SR/SR patients than in the SR/CI patients at the pre-infection time point. Furthermore, this repertoire became more focused at the peak of the immune response during reinfection in patients SR/SR-1 and SR/SR-2 and retained a highly focused composition post-reinfection. This data was confirmed by examining various measures of diversity, richness and evenness. It is possible that those patients, that were recruited as resolvers had previously cleared more than one infection and may have been exposed to more than one genotype, which could have selected the most efficient repertoire. Focusing of the CD8 TCR repertoire upon re-exposure was reported in various infection and vaccination models including LCMV [34]. Focused CD8 TCR repertoires with selection of high-avidity T cell populations were also reported in HIV-1 slow progressors [39]. In contrast, a previous study of an HCV epitope (NS3-1406) suggested that higher diversity of the repertoire would be more advantageous in order to offset viral escape as a result of epitope mutation [20]. A chimpanzee challenge study reached a similar conclusion, since the majority of HCV epitopes that escaped immune recognition upon infection were targeted by a CD8 T cell repertoire with reduced CDR3 amino acid diversity, suggesting that limited TCR diversity facilitates CTL escape mutations in this animal model [17]. Results from the chimpanzee model may not accurately reflect the real life re-exposure for several reasons. First, chimpanzees in these studies were rechallenged with a specific and homologous viral sequence and therefore were exposed to a less diverse viral population as compared to humans that are typically exposed to a complex mixture of viral variants. Second, the chimpanzee data were generated using CD8 T cell clones derived from the livers of infected animals. Examining the CD8 TCR repertoire in the liver of humans is ethically difficult. Although previous analysis demonstrated that PBMCs are a good reflection of the intrahepatic TCR repertoire [21], certain low frequency clonotypes could be enriched within the liver and the *in vitro* expansion step employed in these studies may have introduced some bias in the repertoire.

The Morisita Horn index showed that there were more changes in the repertoire of SR/SR patients upon reinfection (from pre-reinfection to peak reinfection) as compared to SR/CI patients. This could reflect the efficient priming and focusing of the T cell response in SR/SR patients, leading to virus clearance as discussed above. Interestingly, although we could not detect significant expansion of HCV-specific tetramer+ CD8 T cells and associated clonotypes in the SR/CI patients between pre-reinfection and peak, the Morisita Horn index reflected more changes later in reinfection between the peak and late reinfection time points. This suggests that the repertoire has undergone changes following the establishment of chronic infection and the accumulation of viral variants that would prime the expansion of different clonotypes as we also observed that the repertoire became more diverse in those patients (S3 Fig) but obviously this diversification was not enough to clear the virus. Additional analysis accompanied by in depth sequencing of the infecting viral strain at each episode and of instances where the variant epitope is still recognized by the specific T cells are essential to elucidate the interaction between the T cell repertoire, the infecting virus sequence and the emergence of escape mutations.

The presence of a common set of TCRs associated with protection, known as public repertoires, was associated with viral control in several infections including HIV and CMV [5, 6, 40]. Miles and colleagues have also reported a biased TCR repertoire towards a common core of public repertoires in individuals with long-term spontaneous clearance or persistent HCV infection [19]. Our longitudinal analysis of the dominant and subdominant Vβ-CDR3-Jβ clonotypes in the SR/SR and SR/CI patients revealed no overlap between patients with the same HLA background and targeting the same epitope. Given the limited number of patients included in this study, it was not possible to draw a definitive conclusion about whether or not specific public clonotypes were associated with secondary clearance. Additional analysis of a larger cohort of patients targeting the same pMHC is required.

Establishing CD8 T cell lines enabled us to characterize the molecular determinants of functionality of the individual clonotypes. We did not observe different avidity patterns among the 10 cell lines that were analyzed using the tetramer titration assay. Testing more cell lines might be necessary to identify some with a range of TCR avidity as was observed in the HIV and LCMV models [30, 41]. It is also possible that this particular epitope selected mostly CD8 T cells with high avidity and that examining other epitopes may yield different results [42]. Furthermore, this study was performed on individuals that have successfully cleared a previous infection. It is thus possible that the clonotypes that were selected during primary infection and formed the memory pool are those with the highest avidity. Indeed, studies in a mouse model of influenza infection and rechallenge demonstrated that the clonotypes expanding in the recall response were those with the highest avidity [43]. Establishing cell lines specific to the same epitope but from early primary infection samples would thus also be informative. Moreover, a more sensitive method such as surface plasmon resonance might provide a more accurate measure of binding affinity and/or avidity that might be different between cell lines [44].

Functional avidity, or the capacity of a particular clone to translate TCR binding into a functional response, was strikingly different between the cell lines from the SR/SR patient compared to the cell lines from the SR/CI patient, especially for the surface expression of the degranulation marker CD107a and TNFα production. The cell lines established from the SR/SR patient responded well to lower peptide concentration. The polyfunctionality level was also greater in the cell lines from SR/SR as compared to the cell lines from SR/CI and is therefore an important correlate of control of viral replication [8, 45]. We have already demonstrated a broad IFNγ response in the SR/SR-1 patient as measured by ELISPOT assays but the polyfunctional CD8 T cell response to different epitopes was dominated by the production of TNFα and the surface expression of CD107a and that the CD8 T cell response had an increased magnitude and polyfunctionality in the SR/SR patients compared to the SR/CI patients [23]. Hence, the data from the individual cell lines reflected well the overall *in vivo* response. It is possible that in this individual, cytolytic effector functions (CD107a) leading to killing of infected cells provide an overall better antiviral effect as compared to non-cytolytic (IFNγ mediated) functions. Similarly, TNFα systemic levels increase during HCV infection [46] and it can have multiple antiviral and inflammatory effects. Specifically, it can induce the apoptosis of HCV infected hepatocytes and bystander cells in the liver, which could enhance viral clearance [47, 48].

Our study focused on the CD8 T cell response to HCV reinfection. Another important component that remains to be evaluated is the role of the antibody response and the antibody repertoire in protection upon reinfection. Indeed, Osburn et al have demonstrated that reinfection is associated with the development of cross-reactive antibodies [49]. The recent development of novel E2-tetramers that allow sorting and characterization of HCV-specific B cells and antibody repertoire [50] represent an invaluable tool to dissect the role of T cells versus antibodies in protection against reinfection.

In conclusion, our results demonstrate that epitope-specific CD8+ T cell clonotypes expanding at the peak of reinfection are recruited from the memory pool, rather than being *de novo* clonotypes mobilized from the naïve pool. The repertoire is narrower in the SR/SR patients who were protected against viral persistence in comparison to SR/CI patients, and it becomes more focused upon reinfection in 2/3 patients. Analysis of individual CD8 T cell lines from SR/SR versus SR/CI patients revealed that HCV-specific cells associated with resolution of the reinfection had a better functional avidity and polyfunctionality rather than improved avidity of the TCR. Vaccination strategies aiming at enhancing the expansion and polyfunctionality rather than the diversity of HCV-specific T cells by use of adjuvants or immune modulators could be an interesting strategy to follow.

## Materials and methods

### Ethics statement

Study subjects were enrolled among PWIDs participating in the Montreal Acute Hepatitis C Cohort Study (HEPCO) [51]. This study was approved by the Institutional Ethics Committee of CRCHUM (Protocol SL05.014). All samples were anonymized.

### Study population and identification of HCV reinfection cases

Primary HCV infection was identified in HEPCO participants who were initially negative for both HCV RNA and anti-HCV antibodies for at least 6 months, then had a positive HCV RNA and/or antibody test as previously described [32, 51]. Participants who resolved primary HCV infection or participants who tested HCV RNA-negative and HCV antibody-positive at recruitment were enrolled in the reinfection study and followed every 3 months thereafter. HCV reinfection was defined by an HCV-RNA positive test following two negative tests that were performed ≥ 60 days apart. The day of the first positive RNA test was defined as day zero post detection of reinfection. Five cases of reinfection were identified between 2009 and 2012 for whom samples collected prior to reinfection were available. Clinical outcomes and immunological responses in these patients were previously reported [23]. Three patients spontaneously resolved (SR) their second infection (SR/SR group) while two patients became chronically infected (SR/CI group). Patients’ demographics and clinical characteristics are summarized in S1 Table.

### Tetramer staining, flow cytometry and cell sorting

MHC class I tetramers were synthesized by the National Immune Monitoring Laboratory (NIML), (Montréal, QC, Canada) or the NIH Tetramer Core Facility (Emory University, Atlanta, GA). The following tetramers were used: HLA-A1 restricted HCV NS3 peptide amino acids (aa) 1436–1444 (ATDALMTGY) [A1/NS3-1436], HLA-A2 restricted HCV NS3 peptide aa 1073–1081 (CINGVCWTV) [A2/NS3-1073], and HLA-B27 restricted HCV peptide NS5B peptide aa 2841–2849 (ARMILMTHF) [B27/NS5B-2841]. Cryopreserved peripheral blood mononuclear cells (PBMC) were thawed and CD8+ T cells were purified using the negative selection MACS CD8+ T cell Isolation Kit (Miltenyi Biotec Inc, Auburn, CA). Tetramer staining and cell surface staining for CD3, CD8, CD45RO and CD127 were performed as previously described [32]. Directly-conjugated monoclonal antibodies against the following molecules were used: CD3–FITC (clone UCHT1), CD8–Pacific Blue (clone RPA-T8) and CD45RO–Alexa Fluor 700 (clone UCHL1) were obtained from BD Biosciences (San Diego, CA). CD127/IL-7Ra–Alexa Fluor 647 (clone HIL-7R-M21) was obtained from eBioscience (San Diego, CA). Live cells were identified using LIVE/DEAD fixable aqua dead cell stain kit (Molecular Probes Thermo Fisher Scientific, Burlington, ON). Multiparameter flow cytometry was performed on a BD Aria II cell sorter equipped with blue (488 nm), red (633 nm), and violet (405 nm) lasers or a BD LSRII instrument equipped with an additional yellow-green laser (561 nm) using FACSDiva version 6.1.3 (BD Biosciences). Data files were analyzed using FlowJo version 9.4.11 for Mac (Tree Star, Inc., Ashland, OR).

### Sequencing of the TCR β chain

Genomic DNA was extracted from sorted cells and the variable (Vβ), diversity (Dβ) and joining (Jβ) regions of the TCR β chain were sequenced using an automated high-throughput method (Adaptive Biotechnologies, Seattle, WA). Briefly, CDR3 regions were amplified using a two-step amplification bias-controlled multiplex PCR approach [52]. Amplified libraries were sequenced using an Illumina instrument according to the manufacturer’s instructions. Demultiplexed reads were then further processed to reduce amplification and sequencing bias [53]. The resulting CDR3 amino acid sequences were classified into correct families according to the IMGT database (www.imgt.org). Data were analyzed using ImmunoSEQ software (v2.0). Clonotype lists (CDR3 sequences and frequencies within the repertoire) were cleaned to remove out of frame sequences and sequences with stop codons within the CDR3 region. Clonotypes with a frequency of less than 0.01% of the total repertoire were excluded from the analysis since the number of events/sequences would correspond to less than one cell. TCR sequences raw data are available at (https://clients.adaptivebiotech.com/pub/shoukry-2017-plospathogens). S2 Table details the number of sorted cells, total / unique productive sequences and clonality for each sample. Repertoire Simpson diversity index, richness, Shannon entropy index and Morisita Horn index were provided by Adaptive Biotechnologies. Richness index was calculated as the observed richness divided by the input cell number. Evenness was calculated as the Shannon entropy divided by the log of the observed richness. Venn diagrams were generated by comparing the amino acids clonotypes sequences across time points for each patient, after excluding clonotypes with frequencies < 0.01% as explained above. CDR3 length and NT addition for each clonotypes were provided by Adaptive Biotechnologies and the Germline index was calculated as: (Total CDR3 length–total NT additions) / Total CDR3 length.

### Generation of T cell lines

HCV specific, tetramer positive (A2/NS3-1073) CD8+ T cells from two patients (SR/SR-1 and SR/CI-2) were enriched and sorted as described above. Cells were diluted in R10 (RPMI 1640 + 10% heat inactivated fetal bovine serum (FBS; Life Technologies) supplemented with penicillin + streptomycin (pen/strep, 1X, Wisent) and 40U/ml rIL-2 (NIH-AIDS Reagents Program) (R10-P/S-IL2); and plated at a concentration of 5 cells per well in 96 well plates in presence of 5 x 10^4^ non-autologous irradiated (30 Gy) PBMCs as feeder cells and 0.01μg/ml anti-CD3 (Beckman Coulter). Cells were cultured for two weeks in 96 well plates and half of the medium was replenished every 3 days (R10-P/S-IL2). Growing cell lines were then transferred to 24 well plates with a new round of stimulation with feeder cells (2x10^6^ cells/well) and anti-CD3 (0.01μg/ml final). Cell lines were then cryopreserved in freeze mix (FBS + 10% DMSO) at a concentration of 5x10^6^ cells/ml.

### Tetramer titration assay

Avidity of T cell lines was assessed by staining with serial dilutions of tetramers (A2/NS3-1073; 10μg/ml to 0.02μg/ml, two fold dilutions) for 30 min at room temperature in the dark. Surface staining included live/dead marker, CD3-PB (clone UCHT1), CD4-BV605 (clone RPA-T4), CD8-APC-H7 (clone SK1; all from BD Biosciences) and flow cytometry was performed as above.

### Peptide stimulation and Intra-Cytoplasmic Staining (ICS)

T cell lines were stimulated with autologous EBV transformed B cell line (BLCLs) at a ratio of 2:1 (T cell: BLCLs). BLCLs were irradiated at 100 Gy and prepulsed with HCV NS3 peptide (NS3-1073-1081; CINGVCWTV) for 1 h at 37°C in R-10 medium. BLCLs were then washed and incubated with T cell lines for 6 hours in AIM-V medium (Life Technologies) supplemented with 10% human serum (Wisent) and anti-CD107a-BV786 antibody (clone H4A3; BD Bioscience). 10 μg/ml Brefeldin A (BFA, Sigma) and 6 μg/ml monensin (Sigma) were added after 1 h of stimulation. After stimulation, cells were washed and surface staining was performed as described in the tetramer titration section. Cells were then permeabilized with CytoFix/CytoPerm (BD Biosciences) for 15 minutes at 4°C in the dark, washed again, and incubated with anti-IFNγ-PE-Cy7 (clone B27), anti-TNF-α-PerCP-Cy5.5 (clone MAb11) and anti-IL-2-PE (clone MQ1-17H12; all from BD Biosciences) for 30 min at 4°C in the dark. Cells were then washed, fixed and analyzed as above. Polyfunctionality was analyzed using Boolean gating and SPICE software [54].

### Data and materials availability

TCR sequences raw data are available at https://clients.adaptivebiotech.com/pub/shoukry-2017-plospathogens

## Supporting information

S1 Fig The gating strategy used for the sorting of different epitope-specific tetramer+ CD8+ T-cell populations. Pre-purified CD8 T cells were stained and sorted as viable CD3+ CD8+ tetramer positive cells, and when possible also according to the expression of CD127. Naive CD8 T cells were sorted as viable CD3+ CD8+ CD45RO-.
(TIF)

S2 Fig Purity of sorted T-cell populations used for TCR-repertoire analysis. Dot plots showing the post-sorting purity of the sorted (A) epitope-specific tetramer+ CD8+ T-cell population and (B) naïve CD8+ T cells (viable CD3+ CD8+ Tetramer-).
(TIF)

S3 Fig Venn diagrams showing the number of unique clonotypes present for each time point and shared between time points. Clonotypes present only at the pre reinfection time point are located in the red circles, at peak reinfection in the green circle and post reinfection in the blue circle. Clonotypes shared between two or more time points are located at the intersection of the circles. For patient SR/SR-1 the peak reinfection include Effector cells (CD127-) in green and memory cells (CD127+) in yellow.
(TIF)

S4 Fig HCV-specific tetramer+ CD8 T-cell clonotypes recruited during the reinfection episode were present at the peak of primary infection. The top ten dominant clonotypes (frequency ≥1%) isolated directly *ex vivo* from patient SR/SR-3 followed-up longitudinally during primary HCV infection and reinfection episode at pre-reinfection, peak expansion and post reinfection. Tetramer used is indicated between brackets at the top of the graph.
(TIF)

S5 Fig CDR3 sequences characteristics do not differ between groups. Average nucleotides (NT) additions (A), CDR3 region length (B) and germline index (total repertoire (C) and dominant clonotypes only (D)) are shown for both patient groups at the three different time points.
(TIF)

S6 Fig Higher polyfunctionality for resolvers SR/SR compared to chronic SR/CI T cell lines. T cell lines were stimulated with autologous BLCLs prepulsed with increasing concentrations of the cognate peptide (NS3-1073) for 6 h. Surface and intracellular staining was then performed as described in Materials and Methods to examine functionality by flowcytometry. Boolean gating and analysis using spice software was used to assess polyfunctionality profile for each clone established from patient SR/SR-1 (lines R1 to R5) or from patient SR/CI-2 (lines C1 to C5). (A) Representative flow cytometry plot for each function (CD107a; TNFα; IFNγ; IL-2) without (top) and with stimulation (bottom, 10μg/ml peptide). (B-C) T cell clones polyfunctionality represented as pie charts for each T cell line established from HCV resolver (R1 to R5, (B)) or from chronic patient (C1 to C5, (C)). Negative control was T cell lines incubated with BLCL only (no peptide, left). Maximum peptide concentration (10μg/ml, middle and limited concentrations (0.1μg/ml, right) are shown. Data are represented as the percentage of cells with no function (grey); 1 function (yellow); 2 functions (green); 3 functions (orange) and 4 functions (red).
(TIF)

S1 Table Patients’ clinical characteristics and demographics. (DOCX)

S2 Table TCR deep sequencing summary information. (DOCX)

S3 Table Dominant clonotype (Freq ≥1%) usage in A2/NS3-1073 –specific CD8 T cells for patient SR/SR-1 during HCV reinfection. (DOCX)

S4 Table Dominant clonotype (Freq ≥1%) usage in B27/NS5B-2841-specific CD8 T cells for patient SR/SR-2 during HCV reinfection. (DOCX)

S5 Table Dominant clonotype (Freq > 1%) usage in B27/NS5B-2841-specific CD8 T cells for patient SR/SR-3 during HCV reinfection. (DOCX)

S6 Table Dominant clonotype (Freq > 1%) usage in A2/NS3-1073-specific CD8 T cells for patient SR/CI-2 during HCV reinfection. (DOCX)

S7 Table Dominant clonotype (Freq >1%) usage in A1/NS3-1436-specific CD8 T cells for patient SR/CI-3 during HCV reinfection. (DOCX)

S8 Table CD8 T cell lines TCR deep sequencing. (DOCX)
