# Supplementary figures and images for: Selective expansion of high functional avidity memory CD8 T cell clonotypes during hepatitis C virus reinfection and clearance
Source: PLoS Pathog. 2017 Feb 1;13(2):e1006191. doi: 10.1371/journal.ppat.1006191 (PMC5305272; doi:10.1371/journal.ppat.1006191)

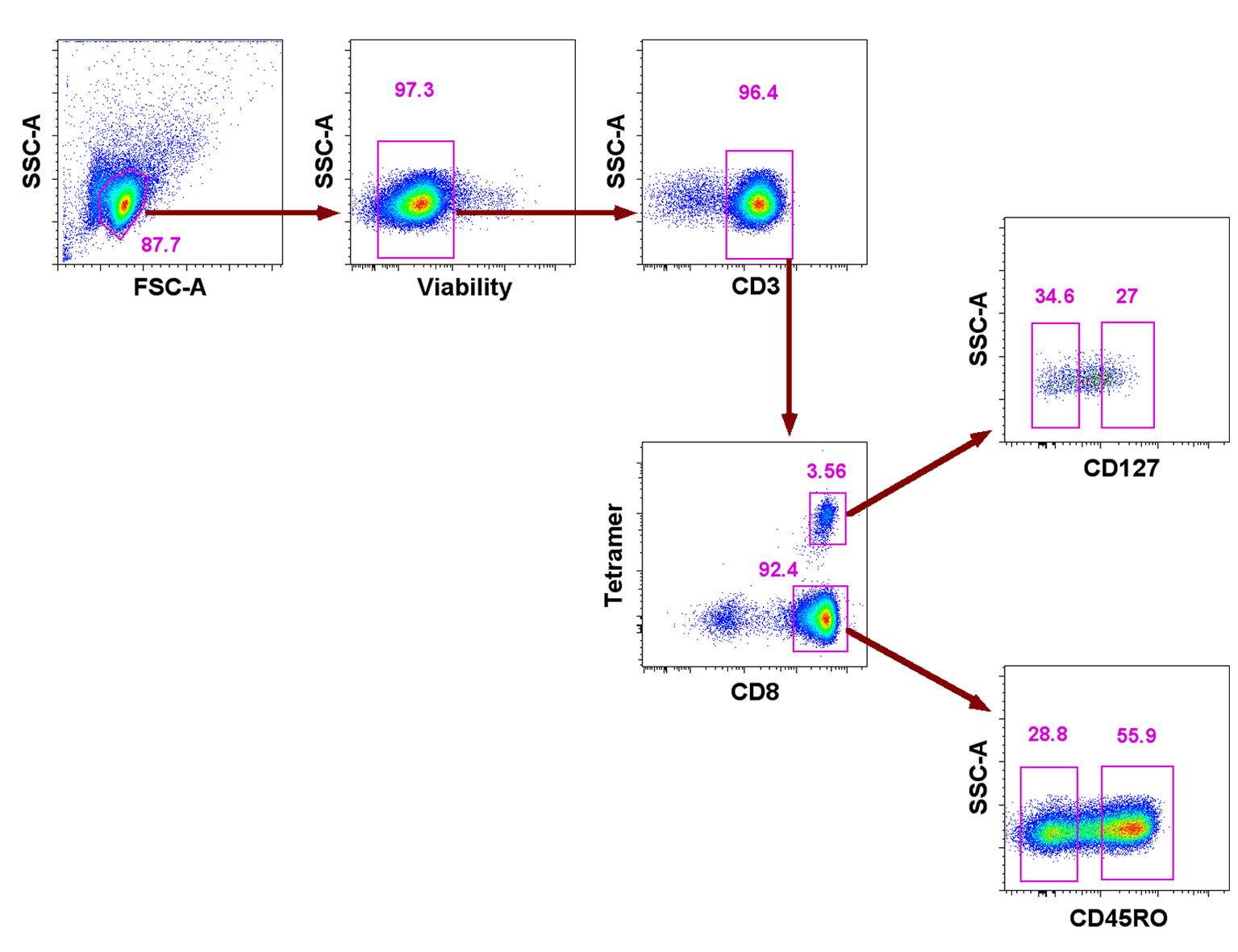

Supplement: S1 Fig — Pre-purified CD8 T cells were stained and sorted as viable CD3+ CD8+ tetramer positive cells, and when possible also according to the expression of CD127. Naive CD8 T cells were sorted as viable CD3+ CD8+ CD45RO-. (TIF) [file ppat.1006191.s001.tif]

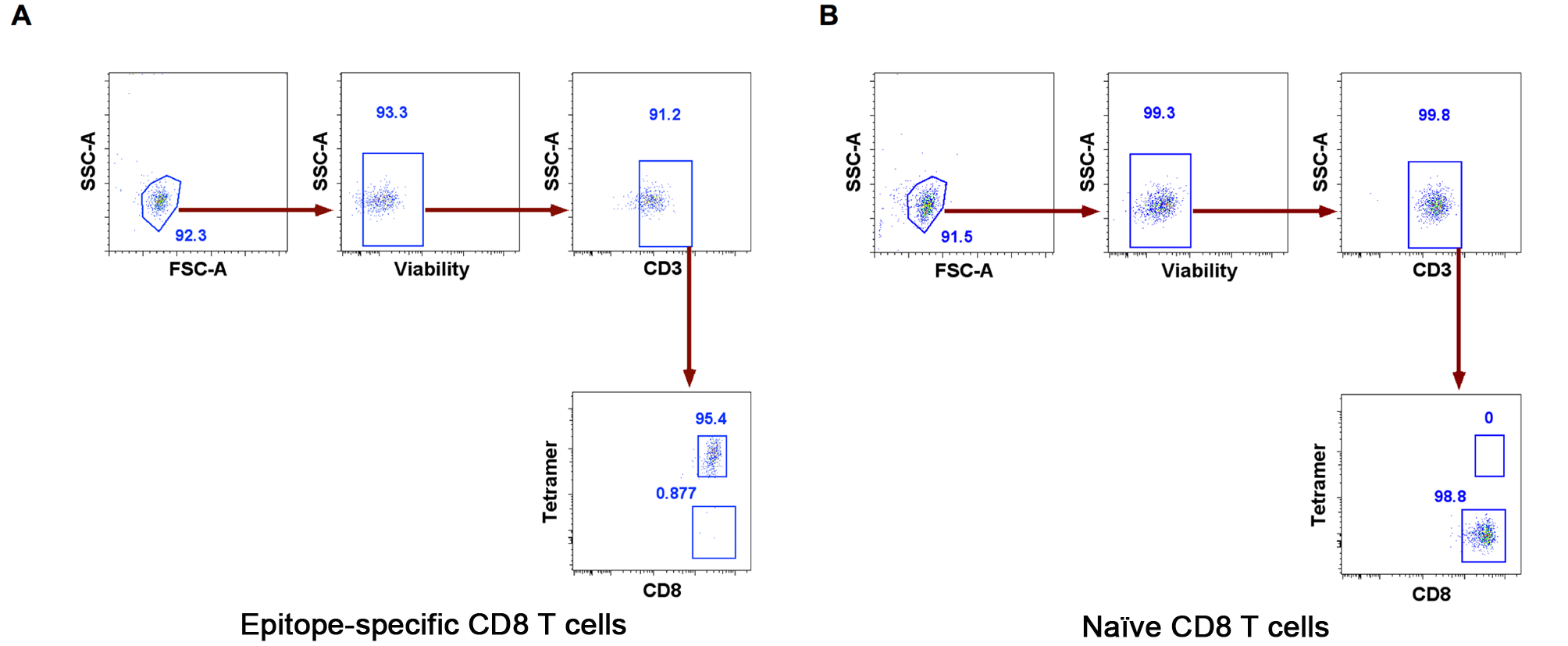

Supplement: S2 Fig — Dot plots showing the post-sorting purity of the sorted (A) epitope-specific tetramer+ CD8+ T-cell population and (B) naïve CD8+ T cells (viable CD3+ CD8+ Tetramer-). (TIF) [file ppat.1006191.s002.tif]

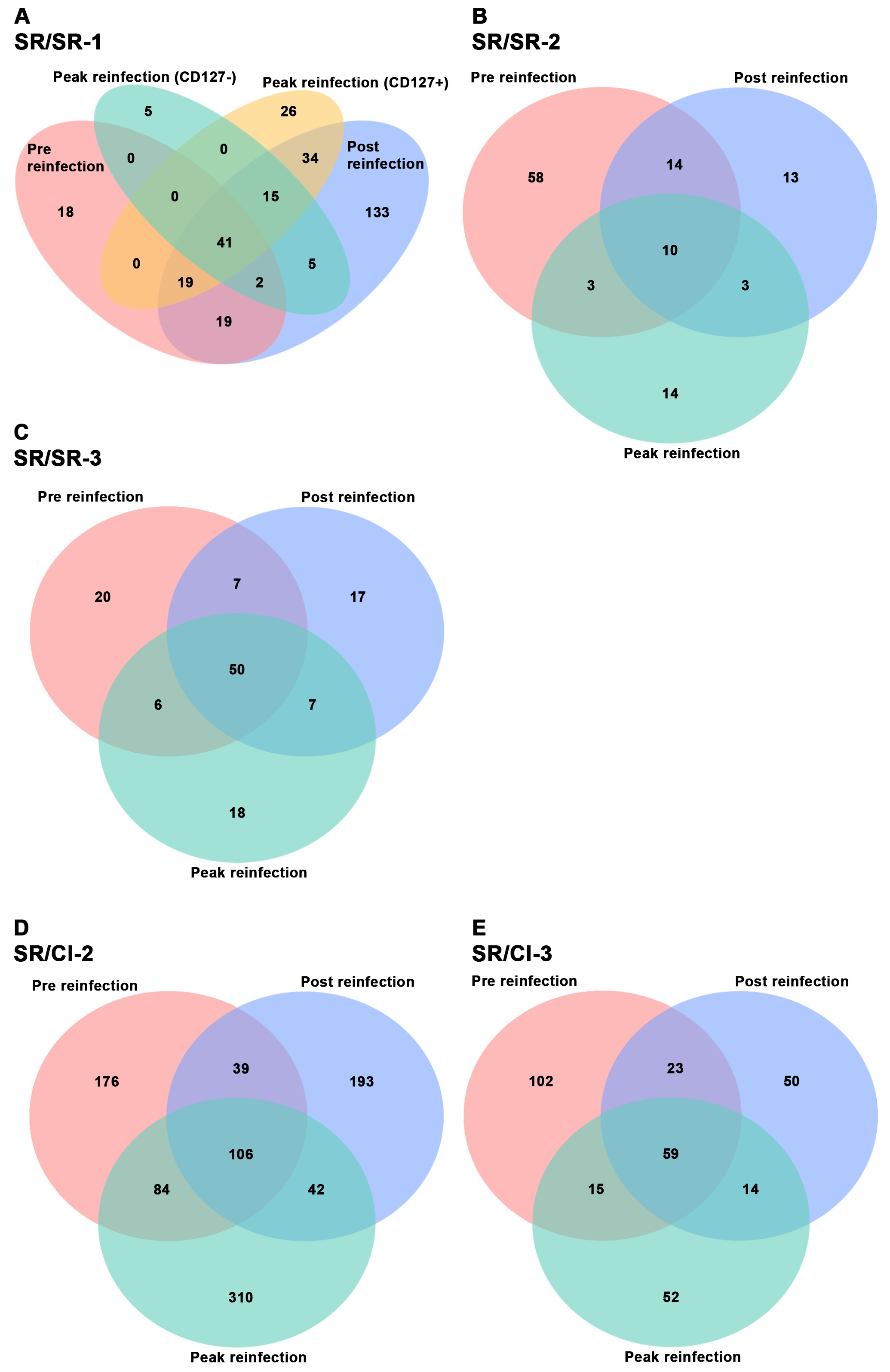

Supplement: S3 Fig — Clonotypes present only at the pre reinfection time point are located in the red circles, at peak reinfection in the green circle and post reinfection in the blue circle. Clonotypes shared between two or more time points are located at the intersection of the circles. For patient SR/SR-1 the peak reinfection include Effector cells (CD127-) in green and memory cells (CD127+) in yellow. (TIF) [file ppat.1006191.s003.tif]

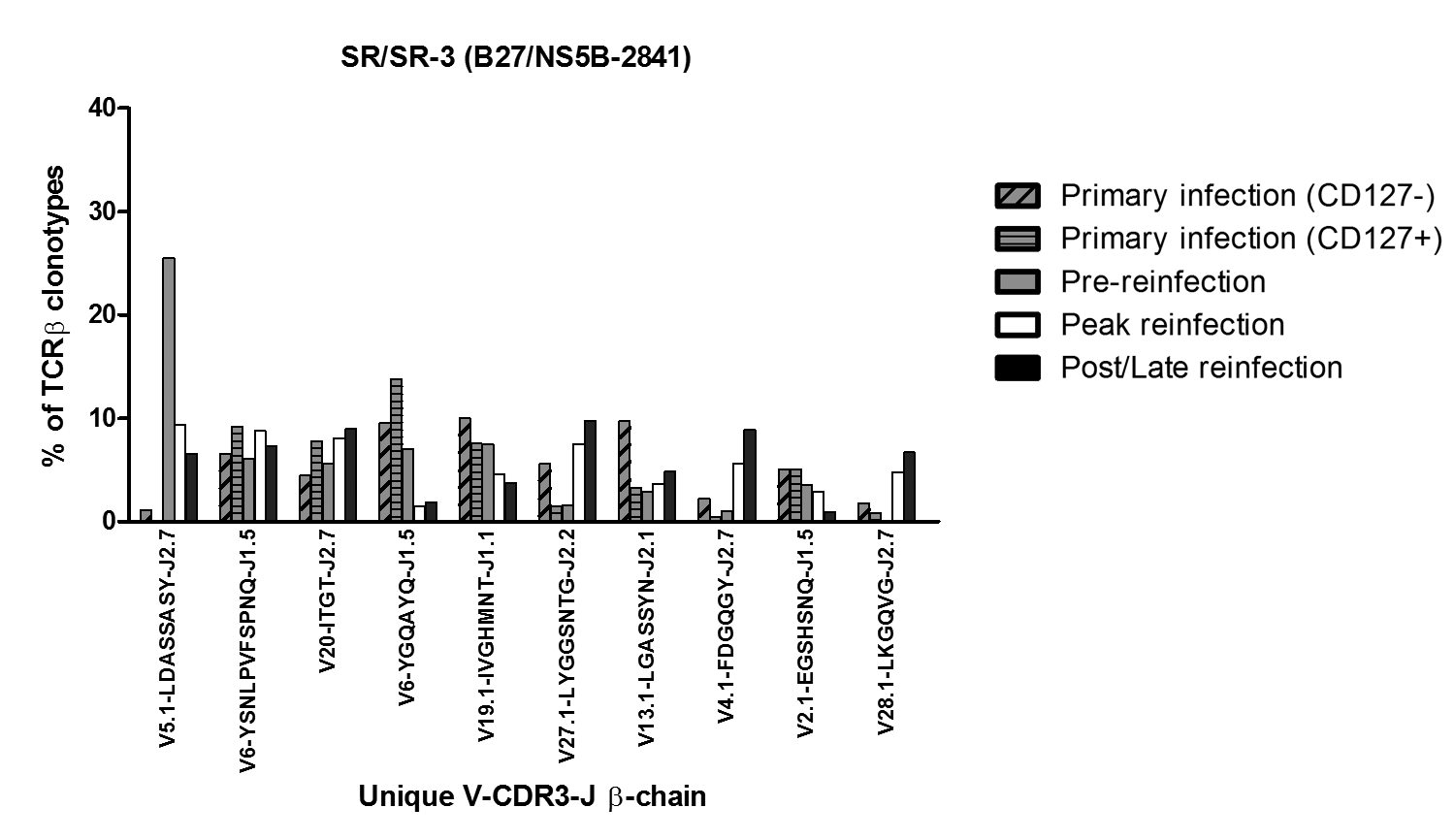

Supplement: S4 Fig — The top ten dominant clonotypes (frequency ≥1%) isolated directly ex vivo from patient SR/SR-3 followed-up longitudinally during primary HCV infection and reinfection episode at pre-reinfection, peak expansion and post reinfection. Tetramer used is indicated between brackets at the top of the graph. (TIF) [file ppat.1006191.s004.tif]

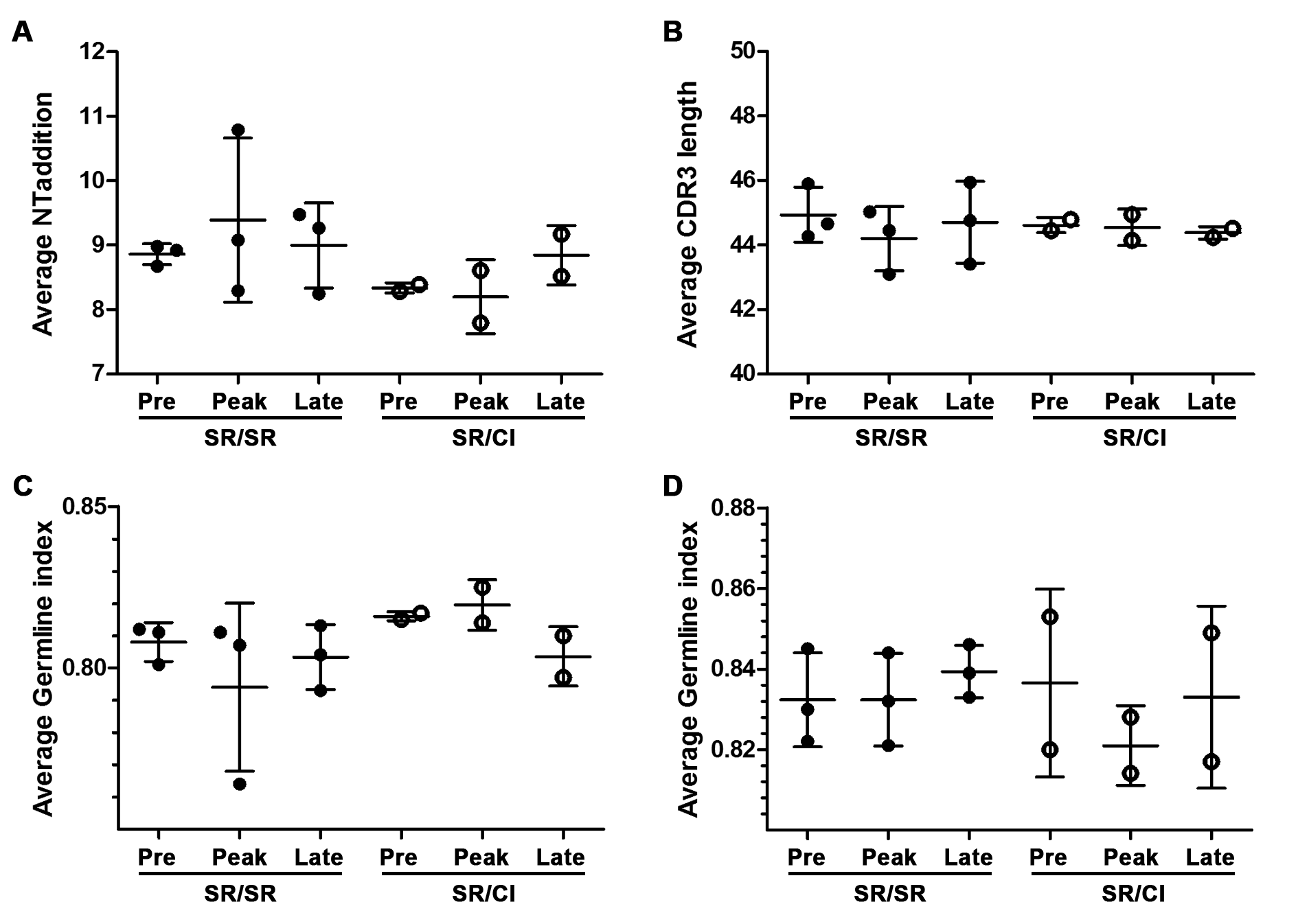

Supplement: S5 Fig — Average nucleotides (NT) additions (A), CDR3 region length (B) and germline index (total repertoire (C) and dominant clonotypes only (D)) are shown for both patient groups at the three different time points. (TIF) [file ppat.1006191.s005.tif]

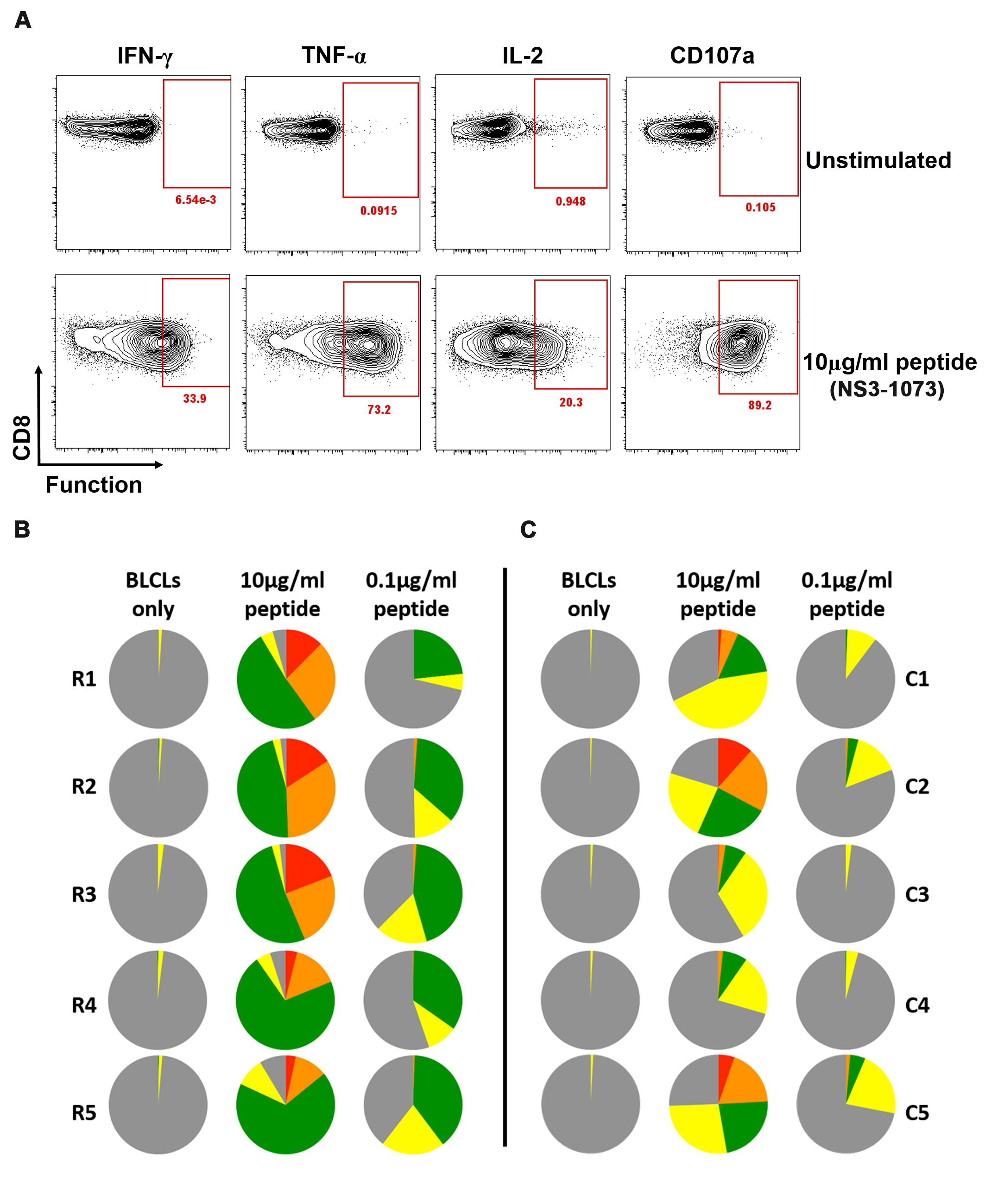

Supplement: S6 Fig — T cell lines were stimulated with autologous BLCLs prepulsed with increasing concentrations of the cognate peptide (NS3-1073) for 6 h. Surface and intracellular staining was then performed as described in Materials and Methods to examine functionality by flowcytometry. Boolean gating and analysis using spice software was used to assess polyfunctionality profile for each clone established from patient SR/SR-1 (lines R1 to R5) or from patient SR/CI-2 (lines C1 to C5). (A) Representative flow cytometry plot for each function (CD107a; TNFα; IFNγ; IL-2) without (top) and with stimulation (bottom, 10μg/ml peptide). (B-C) T cell clones polyfunctionality represented as pie charts for each T cell line established from HCV resolver (R1 to R5, (B)) or from chronic patient (C1 to C5, (C)). Negative control was T cell lines incubated with BLCL only (no peptide, left). Maximum peptide concentration (10μg/ml, middle and limited concentrations (0.1μg/ml, right) are shown. Data are represented as the percentage of cells with no function (grey); 1 function (yellow); 2 functions (green); 3 functions (orange) and 4 functions (red). (TIF) [file ppat.1006191.s006.tif]
