# Supplementary material for: Selective expansion of high functional avidity memory CD8 T cell clonotypes during hepatitis C virus reinfection and clearance
Source: PLoS Pathog. 2017 Feb 1;13(2):e1006191. doi: 10.1371/journal.ppat.1006191 (PMC5305272; doi:10.1371/journal.ppat.1006191)
Supplement: S1 Table — (DOCX) [file ppat.1006191.s007.docx]

**Table S1: Patients’ clinical characteristics and demographics^‡^**

| **Patient ID** | **Age at reinfection (years)** | **Gender** | **HCV Genotype**  **(First infection/**  **reinfection)** | **HLA**  **Class I** | **Tetramers used** | **Time point tested (wks)/ Tetramer frequency**  **(% of CD8+ T cells)** | | | |
| --- | --- | --- | --- | --- | --- | --- | --- | --- | --- |
|  |  |  |  |  |  | **Primary Infection** | **Pre-reinfection** | **Peak reinfection** | **Late reinfection** |
|  |  |  |  |  |  |  |  |  |  |
| **SR/SR-1** | 53 | M | ND/1 | A2, B58 | A2/NS3-1073 | ND | Wk -55  (0.46) | Wk 3  (3.37) | Wk 24  (0.57) |
|  |  |  |  |  |  |  |  |  |  |
| **SR/SR-2** | 43 | M | ND/1 | A24/A26, B27/B49 | B27/NS5B-2841 | ND | Wk -46  (0.16) | Wk 4  (0.25) | Wk 12  (0.15) |
|  |  |  |  |  |  |  |  |  |  |
| **SR/SR-3** | 42 | M | 1a/1 | A23/A32 B27/B44 | B27/NS5B-2841 | Wk 3  (1.0) | Wk -13  (0.57) | Wk 4  (0.30) | Wk 25  (0.44) |
| **SR/CI-2** | 29 | M | 1a/1a | A2, B39/B44 | A2/NS3-1073 | ND | Wk -4  (0.70) | Wk 8  (0.32) | Wk 24  (0.63) |
| **SR/CI-3** | 30 | F | ND/1a | A1/A29 B35/B44 | A1/NS3-1436 | ND | Wk -17  (0.08) | Wk 8  ( 0.10 ) | Wk 18  (0.10) |

ND = Not determined; SR: Spontaneous Resolution; CI: Chronic Infection

**^‡^** The data in this table was previously published in Abdel-Hakeem *et al*. 2014 [23]
