# Supplementary material for: Selective expansion of high functional avidity memory CD8 T cell clonotypes during hepatitis C virus reinfection and clearance
Source: PLoS Pathog. 2017 Feb 1;13(2):e1006191. doi: 10.1371/journal.ppat.1006191 (PMC5305272; doi:10.1371/journal.ppat.1006191)
Supplement: S2 Table — (DOCX) [file ppat.1006191.s008.docx]

**Table S2: TCR deep sequencing summary information**

| **Sample** | **Time point** | **Sorted Cells Number** | **Productive Sequences Total** | **Productive Sequences Unique** | **Clonality** |
| --- | --- | --- | --- | --- | --- |
| **SR/SR-1** | Pre-reinfection | 1 089 | 381 | 118 | 0.2095 |
|  | Peak reinfection (CD127-) | 115 967 | 1 544 | 100 | 0.4316 |
|  | Peak reinfection (CD127+) | 5 813 | 1 781 | 166 | 0.4088 |
|  | Post/Late reinfection | 45 206 | 5 544 | 370 | 0.4088 |
| **SR/SR-2** | Pre-reinfection | 1 054 | 316 | 95 | 0.4125 |
|  | Peak reinfection (total) | 1 604 | 602 | 38 | 0.8024 |
|  | Post/Late reinfection | 1 967 | 494 | 48 | 0.7054 |
| **SR/SR-3** | Primary infection (CD127-) | 4 076 | 1 238 | 150 | 0.2877 |
|  | Primary infection (CD127+) | 6 367 | 992 | 137 | 0.2724 |
|  | Pre-reinfection | 3 302 | 840 | 117 | 0.3276 |
|  | Peak reinfection (total) | 3 237 | 664 | 112 | 0.2509 |
|  | Post/Late reinfection | 2 678 | 584 | 102 | 0.2564 |
| **SR/CI-2** | Pre-reinfection | 1 753 | 1 433 | 528 | 0.1767 |
|  | Peak reinfection (total) | 1 203 | 1 821 | 706 | 0.1631 |
|  | Post/Late reinfection | 1 066 | 993 | 473 | 0.1201 |
| **SR/CI-3** | Pre-reinfection | 6 013 | 470 | 215 | 0.1683 |
|  | Peak reinfection (total) | 3 249 | 323 | 161 | 0.2065 |
|  | Post/Late reinfection | 1 501 | 300 | 154 | 0.1374 |
