# Supplementary material for: Selective expansion of high functional avidity memory CD8 T cell clonotypes during hepatitis C virus reinfection and clearance
Source: PLoS Pathog. 2017 Feb 1;13(2):e1006191. doi: 10.1371/journal.ppat.1006191 (PMC5305272; doi:10.1371/journal.ppat.1006191)
Supplement: S3 Table — (DOCX) [file ppat.1006191.s009.docx]

**Table S3: Dominant clonotype (Freq ≥1%)** **usage in A2/NS3-1073 –specific CD8 T cells for patient SR/SR-1 during HCV reinfection**

| 1. **Patient SR/SR-1 at pre-reinfection (Wk -55)** | | | | |
| --- | --- | --- | --- | --- |
| **TRBV** | **CDR3** | **TRBJ** | **Freq. (%)** | **Count** |
| 06-01 | CASSDPLAGGNEQYF | 02-07 | 11.68 | 7771 |
| 20 | CSAWTGSATEAFF | 01-01 | 8.55 | 5686 |
| 06-01 | CASSEVVTAFSYEQYF | 02-07 | 7 | 4656 |
| 07-09 | CASISSNQPQHF | 01-05 | 4.56 | 3036 |
| 04-02 | CASSQLLAGGSYEQYF | 02-07 | 4 | 2662 |
| 24 | CATSDAPGTGIPYEQYF | 02-07 | 3.78 | 2517 |
| 07-08 | CASSLASLGLALSSYEQYF | 02-07 | 3.71 | 2469 |
| 27-01 | CASSLGTQTYEQYF | 02-07 | 3.16 | 2099 |
| 03 | CASSQDLGLLRDTGELFF | 02-02 | 2.99 | 1986 |
| 27-01 | CASSPGTSDPANYGYTF | 01-02 | 2.69 | 1788 |
| 12 | CASSLSGTGELFF | 02-02 | 2.66 | 1769 |
| 20 | CSAPGPGVSVEKLFF | 01-04 | 2.5 | 1660 |
| 28-01 | CASSSGQGNIQYF | 02-04 | 2.05 | 1366 |
| 03 | CASSQEPSGSWGEQYF | 02-07 | 2.04 | 1360 |
| 04-01 | CASSQAAGVGYPTEAFF | 01-01 | 2.03 | 1350 |
| 10-03 | CAISRDSIQFGNTIYF | 01-03 | 1.99 | 1322 |
| 04-03 | CASSQEQGAPGELFF | 02-02 | 1.77 | 1174 |
| 14-01 | CASSRGPDTEAFF | 01-01 | 1.59 | 1055 |
| 12 | CASSFGDQQYF | 02-07 | 1.4 | 933 |
| 24 | CATSDQSREQYF | 02-07 | 1.34 | 890 |
| 06 | CASSYLFGDANTGELFF | 02-02 | 1.31 | 872 |
| 07-03 | CASSLTCDRTHSVYGYTF | 01-02 | 1.16 | 769 |
| 30-01 | CAWRLGSGEKLFF | 01-04 | 1.12 | 743 |

| 1. **Patient SR/SR-1 at peak reinfection - CD127- Tet+ Cells (Wk 3)** | | | | |
| --- | --- | --- | --- | --- |
| **TRBV** | **CDR3** | **TRBJ** | **Freq. (%)** | **Count** |
| 20 | CSAWTGSATEAFF | 01-01 | 33.37 | 67293 |
| 27-01 | CASSLGTQTYEQYF | 02-07 | 10.12 | 20402 |
| 24 | CATSDAPGTGIPYEQYF | 02-07 | 8.19 | 16511 |
| 27-01 | CASSPGTSDPANYGYTF | 01-02 | 7.54 | 15209 |
| 06-01 | CASSDPLAGGNEQYF | 02-07 | 5.8 | 11692 |
| 07-09 | CASISSNQPQHF | 01-05 | 5.16 | 10411 |
| 28-01 | CASSSGQGNIQYF | 02-04 | 3.57 | 7208 |
| 04-02 | CASSQLLAGGSYEQYF | 02-07 | 3.57 | 7197 |
| 14-01 | CASSRGPDTEAFF | 01-01 | 3.16 | 6376 |
| unresolved | CASSLSGTGELFF | 02-02 | 2.45 | 4947 |
| 28-01 | CASSSGQGNIQYF | 02-04 | 2.37 | 4785 |
| 03 | CASSQDLGLLRDTGELFF | 02-02 | 2.02 | 4069 |
| 20 | CSAQTGRDTEAFF | 01-01 | 1.94 | 3909 |
| 05-06 | CASSQGGDRGDPGDGYTF | 01-02 | 1.23 | 2488 |
| 07-08 | CASSLASLGLALSSYEQYF | 02-07 | 1.09 | 2193 |

| 1. **Patient SR/SR-1 at peak reinfection - CD127+ Tet+ Cells (Wk 3)** | | | | |
| --- | --- | --- | --- | --- |
|  |  |  |  |  |
| **TRBV** | **CDR3** | **TRBJ** | **Freq. (%)** | **Count** |
| 20 | CSAWTGSATEAFF | 01-01 | 25.68 | 57007 |
| 06-01 | CASSDPLAGGNEQYF | 02-07 | 14.86 | 32989 |
| 04 | CATSDAPGTGIPYEQYF | 02-07 | 9.26 | 20554 |
| 27-01 | CASSPGTSDPANYGYTF | 01-02 | 7.86 | 17448 |
| 04-02 | CASSQLLAGGSYEQYF | 02-07 | 4.54 | 10070 |
| 27-01 | CASSLGTQTYEQYF | 02-07 | 3.28 | 7280 |
| 12 | CASSLSGTGELFF | 02-02 | 3.27 | 7249 |
| 06-01 | CASSEVVTAFSYEQYF | 02-07 | 2.63 | 5831 |
| 03 | CASSQDLGLLRDTGELFF | 02-02 | 2.53 | 5614 |
| 07-09 | CASISSNQPQHF | 01-05 | 2.2 | 4875 |
| 03 | CASSQEPSGSWGEQYF | 02-07 | 1.86 | 4133 |
| 07-08 | CASSLASLGLALSSYEQYF | 02-07 | 1.26 | 2787 |
| 14-01 | CASSRGPDTEAFF | 01-01 | 1.14 | 2540 |
| 28-01 | CASSSGQGNIQYF | 02-04 | 1.07 | 2366 |

| 1. **Patient SR/SR-1 at post reinfection (Wk 24)** | | | | |
| --- | --- | --- | --- | --- |
| **TRBV** | **CDR3** | **TRBJ** | **Freq. (%)** | **Count** |
| 20 | CSAWTGSATEAFF | 01-01 | 15.62 | 78987 |
| 06-01 | CASSDPLAGGNEQYF | 02-07 | 13.45 | 68019 |
| 24 | CATSDAPGTGIPYEQYF | 02-07 | 10.14 | 51281 |
| 27-01 | CASSPGTSDPANYGYTF | 01-02 | 7.25 | 36680 |
| 27-01 | CASSLGTQTYEQYF | 02-07 | 5.25 | 26560 |
| 04-02 | CASSQLLAGGSYEQYF | 02-07 | 4.98 | 25169 |
| 07-09 | CASISSNQPQHF | 01-05 | 3.56 | 17975 |
| 12 | CASSLSGTGELFF | 02-02 | 2.94 | 14843 |
| 06-01 | CASSEVVTAFSYEQYF | 02-07 | 2.28 | 11524 |
| 07-08 | CASSLASLGLALSSYEQYF | 02-07 | 2.19 | 11083 |
| 28-01 | CASSSGQGNIQYF | 02-04 | 1.89 | 9543 |
| 03 | CASSQDLGLLRDTGELFF | 02-02 | 1.74 | 8775 |
| 14-01 | CASSRGPDTEAFF | 01-01 | 1.69 | 8569 |
| 04-01 | CASSQAAGVGYPTEAFF | 01-01 | 1.62 | 8186 |
| 03 | CASSQEPSGSWGEQYF | 02-07 | 1.51 | 7629 |
| 05-06 | CASSQGGDRGDPGDGYTF | 01-02 | 1.15 | 5800 |
