# Supplementary material for: Selective expansion of high functional avidity memory CD8 T cell clonotypes during hepatitis C virus reinfection and clearance
Source: PLoS Pathog. 2017 Feb 1;13(2):e1006191. doi: 10.1371/journal.ppat.1006191 (PMC5305272; doi:10.1371/journal.ppat.1006191)
Supplement: S4 Table — (DOCX) [file ppat.1006191.s010.docx]

**Table S4: Dominant clonotype (Freq ≥1%) usage in B27/NS5B-2841-specific CD8 T cells for patient SR/SR-2 during HCV reinfection**

| 1. **Patient SR/SR-2 at pre-reinfection (Wk -46)** | | | | |
| --- | --- | --- | --- | --- |
| **TRBV** | **CDR3** | **TRBJ** | **Freq. (%)** | **Count** |
| 11-02 | CASSFDSGAYEQYF | 02-07 | 35.97 | 13631 |
| 19-01 | CASSTKQGWYEQYF | 02-07 | 23.46 | 8889 |
| 10-02 | CASSDAYNEKLFF | 01-04 | 2.9 | 1098 |
| 19-01 | CASSTKQGWYEQYF | 02-07 | 2.81 | 1065 |
| 04-01 | CASSPLVQGGLQPQHF | 01-05 | 1.9 | 720 |
| 02-01 | CASSEGTTNTGELFF | 02-02 | 1.52 | 575 |
| 07-03 | CASSWDSGAEAFF | 01-01 | 1.45 | 549 |
| 05-06 | CASSLKAVVTGELFF | 02-02 | 1.44 | 544 |
| 04-01 | CASSFDGGNQPQHF | 01-05 | 1.3 | 493 |
| 06 | CASSIDKLNTEAFF | 01-01 | 1.22 | 464 |
| 04-01 | CASSFDFQGSYEQYF | 02-07 | 1.16 | 439 |
| 04-01 | CASSQVHLGQSTLNTEAFF | 01-01 | 1.12 | 425 |
| 06 | CASALRGANVLTF | 02-06 | 1.07 | 404 |
| 27-01 | CASSPYGGSNTGELFF | 02-02 | 1.05 | 397 |
|  |  |  |  |  |
| 1. **Patient SR/SR-2 at peak reinfection (Wk 4)** | | | | |
| **TRBV** | **CDR3** | **TRBJ** | **Freq. (%)** | **Count** |
| 19-01 | CASSTKQGWYEQYF | 02-07 | 81.88 | 68836 |
| 11-02 | CASSFDSGAYEQYF | 02-07 | 13.7 | 11514 |
|  |  |  |  |  |
| 1. **Patient SR/SR-2 at post reinfection (Wk 12)** | | | | |
| **TRBV** | **CDR3** | **TRBJ** | **Freq. (%)** | **Count** |
| 19-01 | CASSTKQGWYEQYF | 02-07 | 66.44 | 40660 |
| 11-02 | CASSFDSGAYEQYF | 02-07 | 24.29 | 14863 |
| 04-01 | CASSFDGGNQPQHF | 01-05 | 1.07 | 656 |
| 10-02 | CASSDAYNEKLFF | 01-04 | 1.02 | 625 |
