# Supplementary material for: Selective expansion of high functional avidity memory CD8 T cell clonotypes during hepatitis C virus reinfection and clearance
Source: PLoS Pathog. 2017 Feb 1;13(2):e1006191. doi: 10.1371/journal.ppat.1006191 (PMC5305272; doi:10.1371/journal.ppat.1006191)
Supplement: S5 Table — (DOCX) [file ppat.1006191.s011.docx]

**Table S5: Dominant clonotype (Freq > 1%) usage in B27/NS5B-2841-specific CD8 T cells for patient SR/SR-3 during HCV reinfection**

| 1. **Patient SR/SR-3 at primary infection - CD127- Tet+ Cells (Wk -53)** | | | |
| --- | --- | --- | --- |
| **CDR3** | **TRBJ** | **Freq. (%)** | **Count** |
| CASRLGASSYNEQFF | 02-01 | 9.83 | 17131 |
| CASSYGQAYQPQHF | 01-05 | 9.56 | 16650 |
| CASSYSNLPVFSPNQPQHF | 01-05 | 6.58 | 11465 |
| CASSIVGHMNTEAFF | 01-01 | 6.06 | 10549 |
| CASSLYGGSNTGELFF | 02-02 | 5.62 | 9791 |
| CASSEGSHSNQPQHF | 01-05 | 5.15 | 8966 |
| CSASITGTEQYF | 02-07 | 4.51 | 7852 |
| CASSIVGHLNTEAFF | 01-01 | 3.87 | 6737 |
| CASSRARGSTEVREQFF | 02-01 | 3.8 | 6619 |
| CASGYAGANVLTF | 02-06 | 3.47 | 6042 |
| CASSFDRGGYEQYF | 02-07 | 2.56 | 4451 |
| CASSFDGQGYEQYF | 02-07 | 2.2 | 3833 |
| CASSPATGTEAFF | 01-01 | 2.18 | 3799 |
| CASSLNLGAGNNEQFF | 02-01 | 2.1 | 3657 |
| CSVGGTGTYGYTF | 01-02 | 2 | 3486 |
| CASSLKGQVGEQYF | 02-07 | 1.77 | 3079 |
| CASSVSPDNTDTQYF | 02-03 | 1.48 | 2571 |
| CASSLEGSAGELFF | 02-02 | 1.42 | 2469 |
| CAISESGQALITYEQYF | 02-07 | 1.42 | 2468 |
| CASSPGTTNTGELFF | 02-02 | 1.33 | 2323 |
| CASSLASNEQFF | 02-01 | 1.33 | 2312 |
| CASSHDTGGYEQYF | 02-07 | 1.26 | 2202 |
| CASSFLGGLDEQFF | 02-01 | 1.21 | 2113 |
| CASSFDRGGYEQYF | 02-07 | 1.21 | 2110 |
| CASSLDASSASYEQYF | 02-07 | 1.13 | 1974 |
| CASSSGLALVFEQFF | 02-01 | 1.1 | 1910 |
| CASSFSSGGYEQYF | 02-07 | 1.04 | 1813 |

| 1. **Patient SR/SR-3 at primary infection - CD127+ Tet+ Cells (Wk -53)** | | | | |
| --- | --- | --- | --- | --- |
| **TRBV** | **CDR3** | **TRBJ** | **Freq. (%)** | **Count** |
| 06 | CASSYGQAYQPQHF | 01-05 | 13.78 | 16767 |
| 06 | CASSYSNLPVFSPNQPQHF | 01-05 | 9.21 | 11203 |
| 20 | CSASITGTEQYF | 02-07 | 7.78 | 9466 |
| 19-01 | CASSIVGHLNTEAFF | 01-01 | 5.46 | 6638 |
| 02-01 | CASSEGSHSNQPQHF | 01-05 | 5.08 | 6184 |
| 13-01 | CASRLGASSYNEQFF | 02-01 | 3.3 | 4013 |
| 04-01 | CASTAGSNTGELFF | 02-02 | 2.7 | 3288 |
| 29-01 | CSVGGTGTYGYTF | 01-02 | 2.64 | 3210 |
| 05-06 | CASSLNLGAGNNEQFF | 02-01 | 2.49 | 3030 |
| 11-02 | CASSFLGGLDEQFF | 02-01 | 2.4 | 2922 |
| 28-01 | CASSLEGSAGELFF | 02-02 | 2.2 | 2678 |
| 05-01 | CASSFDRGGYEQYF | 02-07 | 2.08 | 2531 |
| 11-02 | CASSLAVSSEQFF | 02-01 | 2.07 | 2524 |
| 19-01 | CASSIVGHMNTEAFF | 01-01 | 2.07 | 2516 |
| 05-06 | CASSLGLPGLAGAYEQYF | 02-07 | 2.03 | 2475 |
| 04-01 | CASSFDTGGYEQYF | 02-07 | 1.93 | 2345 |
| 06 | CASSWTAIEQYF | 02-07 | 1.77 | 2159 |
| 18-01 | CASSPATGTEAFF | 01-01 | 1.77 | 2148 |
| 05-01 | CASSPGTTNTGELFF | 02-02 | 1.7 | 2074 |
| 06-05 | CASSSGLALVFEQFF | 02-01 | 1.61 | 1953 |
| 11-02 | CASSLASNEQFF | 02-01 | 1.55 | 1890 |
| 27-01 | CASSLYGGSNTGELFF | 02-02 | 1.47 | 1792 |
| 12-05 | CASGYAGANVLTF | 02-06 | 1.35 | 1644 |
| 10-03 | CAISESGQALITYEQYF | 02-07 | 1.25 | 1519 |
| 04-03 | CASSRARGSTEVREQFF | 02-01 | 1.17 | 1427 |
| 04-03 | CASSFDRGGYEQYF | 02-07 | 1.08 | 1316 |

| 1. **Patient SR/SR-3 at pre-reinfection (Wk -34)** | | | | |
| --- | --- | --- | --- | --- |
| **TRBV** | **CDR3** | **TRBJ** | **Freq. (%)** | **Count** |
| 05-01 | CASSLDASSASYEQYF | 02-07 | 25.51 | 23533 |
| 05-01 | CASREDGGGYEQYF | 02-07 | 7.03 | 6486 |
| 06 | CASSYGQAYQPQHF | 01-05 | 6.96 | 6418 |
| 06 | CASSYSNLPVFSPNQPQHF | 01-05 | 6.07 | 5597 |
| 20 | CSASITGTEQYF | 02-07 | 5.55 | 5122 |
| 19-01 | CASSIVGHLNTEAFF | 01-01 | 4.23 | 3898 |
| 02-01 | CASSEGSHSNQPQHF | 01-05 | 3.56 | 3279 |
| 19-01 | CASSIVGHMNTEAFF | 01-01 | 3.25 | 3002 |
| 13-01 | CASRLGASSYNEQFF | 02-01 | 2.86 | 2637 |
| 05-06 | CASSLNLGAGNNEQFF | 02-01 | 2.69 | 2478 |
| 28-01 | CASSLEGSAGELFF | 02-02 | 2.61 | 2411 |
| 27-01 | CASSLYGGSNTGELFF | 02-02 | 1.62 | 1496 |
| 29-01 | CSVGGTGTYGYTF | 01-02 | 1.51 | 1391 |
| 27-01 | CASSLYRVGYNEQFF | 02-01 | 1.48 | 1366 |
| 11-02 | CASSFLGGLDEQFF | 02-01 | 1.44 | 1328 |
| 05-01 | CASSPGTTNTGELFF | 02-02 | 1.36 | 1251 |
| 05-01 | CASSFDRGGYEQYF | 02-07 | 1.32 | 1218 |
| 05-06 | CASSLGLPGLAGAYEQYF | 02-07 | 1.21 | 1113 |
| 10-02 | CASCDNTGYEQYF | 02-07 | 1.06 | 974 |
| 11-02 | CASSLAVSSEQFF | 02-01 | 1.03 | 953 |
| 04-01 | CASSFDGQGYEQYF | 02-07 | 1.03 | 952 |
|  |  |  |  |  |

| 1. **Patient SR/SR-3 at peak reinfection (Wk 4)** | | | | |
| --- | --- | --- | --- | --- |
| **TRBV** | **CDR3** | **TRBJ** | **Freq. (%)** | **Count** |
| 05-01 | CASSLDASSASYEQYF | 02-07 | 9.43 | 7774 |
| 06 | CASSYSNLPVFSPNQPQHF | 01-05 | 8.81 | 7265 |
| 20 | CSASITGTEQYF | 02-07 | 8.08 | 6662 |
| 27-01 | CASSLYGGSNTGELFF | 02-02 | 7.46 | 6150 |
| 04-01 | CASSFDGQGYEQYF | 02-07 | 5.63 | 4644 |
| 28-01 | CASSLKGQVGEQYF | 02-07 | 4.77 | 3931 |
| 13-01 | CASRLGASSYNEQFF | 02-01 | 3.7 | 3049 |
| 05-01 | CASSFDRGGYEQYF | 02-07 | 3.56 | 2933 |
| 19-01 | CASSIVGHLNTEAFF | 01-01 | 3.49 | 2878 |
| 05-01 | CASREDGGGYEQYF | 02-07 | 3.44 | 2840 |
| 28-01 | CASSLEGSAGELFF | 02-02 | 3.16 | 2606 |
| 02-01 | CASSEGSHSNQPQHF | 01-05 | 2.88 | 2373 |
| 04-03 | CASSRARGSTEVREQFF | 02-01 | 2.31 | 1905 |
| 27-01 | CASSLYRVGYNEQFF | 02-01 | 2.07 | 1711 |
| 29-01 | CSVGGTGTYGYTF | 01-02 | 1.86 | 1533 |
| 11-02 | CASSLAVSSEQFF | 02-01 | 1.83 | 1513 |
| 11-02 | CASSLASNEQFF | 02-01 | 1.81 | 1494 |
| 04-03 | CASSQSSMQDPYGYTF | 01-02 | 1.55 | 1278 |
| 06 | CASSYGQAYQPQHF | 01-05 | 1.5 | 1235 |
| 04-03 | CASSFDRGGYEQYF | 02-07 | 1.39 | 1144 |
| 06 | CASSWTAIEQYF | 02-07 | 1.19 | 982 |
| 19-01 | CASSIVGHMNTEAFF | 01-01 | 1.1 | 909 |
| 02-01 | CASNFDKGGYEQYF | 02-07 | 1.07 | 885 |
| 09-01 | CASSVSPDNTDTQYF | 02-03 | 1.06 | 871 |
| 29-01 | CSVAHTGTEQYF | 02-07 | 1.03 | 847 |
|  |  |  |  |  |

| 1. **Patient SR/SR-3 at post reinfection (Wk 12)** | | | | |
| --- | --- | --- | --- | --- |
| **TRBV** | **CDR3** | **TRBJ** | **Freq. (%)** | **Count** |
| 27-01 | CASSLYGGSNTGELFF | 02-02 | 9.75 | 7078 |
| 20 | CSASITGTEQYF | 02-07 | 9.04 | 6562 |
| 04-01 | CASSFDGQGYEQYF | 02-07 | 8.87 | 6438 |
| V06 | CASSYSNLPVFSPNQPQHF | 01-05 | 7.31 | 5309 |
| 28-01 | CASSLKGQVGEQYF | 02-07 | 6.71 | 4870 |
| 05-01 | CASSLDASSASYEQYF | 02-07 | 6.56 | 4762 |
| 13-01 | CASRLGASSYNEQFF | 02-01 | 4.86 | 3525 |
| 05-01 | CASSFDRGGYEQYF | 02-07 | 3.85 | 2792 |
| 05-01 | CASREDGGGYEQYF | 02-07 | 3.51 | 2546 |
| 28-01 | CASSLEGSAGELFF | 02-02 | 2.87 | 2085 |
| 19-01 | CASSIVGHLNTEAFF | 01-01 | 2.5 | 1813 |
| 04-03 | CASSQSSMQDPYGYTF | 01-02 | 2.41 | 1750 |
| 27-01 | CASSLYRVGYNEQFF | 02-01 | 2.29 | 1663 |
| 11-02 | CASSLASNEQFF | 02-01 | 2.22 | 1614 |
| 09-01 | CASSVSPDNTDTQYF | 02-03 | 2.01 | 1461 |
| 06 | CASSYGQAYQPQHF | 01-05 | 1.89 | 1374 |
| 11-02 | CASSLAVSSEQFF | 02-01 | 1.72 | 1252 |
| 29-01 | CSVGGTGTYGYTF | 01-02 | 1.47 | 1068 |
| 19-01 | CASSIVGHMNTEAFF | 01-01 | 1.26 | 917 |
