# Supplementary material for: Selective expansion of high functional avidity memory CD8 T cell clonotypes during hepatitis C virus reinfection and clearance
Source: PLoS Pathog. 2017 Feb 1;13(2):e1006191. doi: 10.1371/journal.ppat.1006191 (PMC5305272; doi:10.1371/journal.ppat.1006191)
Supplement: S6 Table — (DOCX) [file ppat.1006191.s012.docx]

**Table S6: Dominant clonotype (Freq > 1%) usage in A2/NS3-1073-specific CD8 T cells for patient SR/CI-2 during HCV reinfection**

| 1. **Patient SR/CI-2 at pre-reinfection (Wk -20)** | | | | |
| --- | --- | --- | --- | --- |
| **TRBV** | **CDR3** | **TRBJ** | **Freq. (%)** | **Count** |
| 06 | CASTSQSELFF | 01-04 | 6.91 | 21786 |
| 03 | CASSRFLGSGGAAEQFF | 02-01 | 4.22 | 13302 |
| 07-09 | CASLEEDTGELFF | 02-02 | 4.13 | 13023 |
| 05-06 | CASGTGANEKLFF | 01-04 | 2.85 | 8983 |
| 06-05 | CASIRLAGEHNEQFF | 02-01 | 2.77 | 8742 |
| 04-03 | CASSQDPLAGGGGEQFF | 02-01 | 1.71 | 5397 |
| 02-01 | CASSDGQTEKLFF | 01-04 | 1.62 | 5105 |
| 24 | CANRDRGRDEQFF | 02-01 | 1.54 | 4860 |
| 07-06 | CASSLEVAGGNEQFF | 02-01 | 1.38 | 4360 |
| 06-05 | CASVGGNYGYTF | 01-02 | 1.36 | 4282 |
| 06-06 | CASSYSAGTLDYGYTF | 01-02 | 1.32 | 4149 |
| 04-02 | CASSQQGEKLFF | 01-04 | 1.24 | 3912 |
| 02-01 | CASSQGQTNEKLFF | 01-04 | 1.14 | 3605 |
| 05-06 | CASSTGGGTEAFF | 01-01 | 1.11 | 3505 |
| 06-01 | CASSELGTGDYEQYF | 02-07 | 1.06 | 3341 |
| 05-04 | CASSLPQGRYMNTEAFF | 01-01 | 1.04 | 3286 |
| 02-01 | CASRDVVGRLSSYNEQFF | 02-01 | 1.02 | 3201 |
| 06-06 | CASTRDTEAFF | 01-01 | 1 | 3168 |
|  |  |  |  |  |
|  |  |  |  |  |

| 1. **Patient SR/CI-2 at peak reinfection (Wk 10)** | | | | |
| --- | --- | --- | --- | --- |
| **TRBV** | **CDR3** | **TRBJ** | **Freq. (%)** | **Count** |
| 06 | CASTSQSELFF | 01-04 | 6.47 | 30139 |
| 03 | CASSRFLGSGGAAEQFF | 02-01 | 5.44 | 25332 |
| 06-05 | CASIRLAGEHNEQFF | 02-01 | 2.86 | 13300 |
| 07-09 | CASLEEDTGELFF | 02-02 | 2.68 | 12479 |
| 04-03 | CASSQDPLAGGGGEQFF | 02-01 | 1.91 | 8907 |
| 04-02 | CASSPEQGPLEAFF | 01-01 | 1.42 | 6618 |
| 24 | CANRDRGRDEQFF | 02-01 | 1.31 | 6083 |
| 06 | CASSPWAVGAMNTEAFF | 01-01 | 1.31 | 6082 |
| 06-01 | CASSELGTGDYEQYF | 02-07 | 1.3 | 6074 |
| 05-06 | CASGTGANEKLFF | 01-04 | 1.28 | 5974 |
| 02-01 | CASSDGQTEKLFF | 01-04 | 1.25 | 5833 |
| 02-01 | CASRDVVGRLSSYNEQFF | 02-01 | 1.11 | 5152 |
|  |  |  |  |  |
| 1. **Patient SR/CI-2 at late reinfection (Wk 66)** | | | | |
| **TRBV** | **CDR3** | **TRBJ** | **Freq. (%)** | **Count** |
| 04-01 | CASSQGLPNEKLFF | 01-04 | 3.18 | 8151 |
| 02-01 | CASSQGQTNEKLFF | 01-04 | 2.68 | 6872 |
| 05-04 | CASSLEQGAVDGNTIYF | 01-03 | 2.63 | 6745 |
| 06-05 | CASIRLAGEHNEQFF | 02-01 | 2.18 | 5584 |
| 04-03 | CASSQDPLAGGGGEQFF | 02-01 | 2.15 | 5520 |
| 07-06 | CASSLEVAGGNEQFF | 02-01 | 1.99 | 5100 |
| 03 | CASSRFLGSGGAAEQFF | 02-01 | 1.7 | 4353 |
| 02-01 | CASSQGQTNEKLFF | 01-04 | 1.62 | 4155 |
| 05-06 | CASSLRSDTEAFF | 01-01 | 1.38 | 3530 |
| 21-01 | CASSKQTGTGETKNIQYF | 02-04 | 1.37 | 3514 |
| 19-01 | CASSTPPGVNSNQPQHF | 01-05 | 1.29 | 3313 |
| 02-01 | CASRDVVGRLSSYNEQFF | 02-01 | 1.22 | 3123 |
| 19-01 | CASGWPGGLNSNQPQHF | 01-05 | 1.09 | 2798 |
| 06-06 | CASSSAPEGLSYEQYF | 02-07 | 1 | 2567 |
