# Supplementary material for: Selective expansion of high functional avidity memory CD8 T cell clonotypes during hepatitis C virus reinfection and clearance
Source: PLoS Pathog. 2017 Feb 1;13(2):e1006191. doi: 10.1371/journal.ppat.1006191 (PMC5305272; doi:10.1371/journal.ppat.1006191)
Supplement: S7 Table — (DOCX) [file ppat.1006191.s013.docx]

**Table S7: Dominant clonotype (Freq >1%) usage in A1/NS3-1436-specific CD8 T cells for patient SR/CI-3 during HCV reinfection**

|  |  |  |  |  |
| --- | --- | --- | --- | --- |
| 1. **Patient SR/CI-3 at pre-reinfection (Wk -37)** | | | | |
| **TRBV** | **CDR3** | **TRBJ** | **Freq. (%)** | **Count** |
| 10-03 | CAISESTAGSQPQHF | 01-05 | 10.65 | 4989 |
| 07-09 | CAGNNRDSSTDTQYF | 02-03 | 5.34 | 2501 |
| 10-03 | CAITDGASGANEQFF | 02-01 | 4.83 | 2263 |
| 11-02 | CASSLILAGGSYNEQFF | 02-01 | 3.53 | 1656 |
| 10-03 | CAISESPQSYEQYF | 02-07 | 2.3 | 1077 |
| 20 | CSARPIDSSNQPQHF | 01-05 | 2.25 | 1056 |
| 02-01 | CASSEGEINNQPQHF | 01-05 | 2.23 | 1047 |
| 05-01 | CASRGDTFYEQYF | 02-07 | 2.17 | 1019 |
| 07-03 | CASSLVGSGDTQYF | 02-03 | 2.17 | 1016 |
| 12 | CASSIAGPPYNEQFF | 02-01 | 1.83 | 856 |
| 24 | CATSDASLSSYNEQFF | 02-01 | 1.77 | 831 |
| 28-01 | CASRTPGHLYEQYF | 02-07 | 1.75 | 818 |
| 02-01 | CASSEIGQLETQYF | 02-05 | 1.66 | 780 |
| 05-01 | CASSWWETSHEQFF | 02-01 | 1.51 | 706 |
| 09-01 | CASSPASSSFYEQYF | 02-07 | 1.46 | 685 |
| 19-01 | CASSIRDIGNQPQHF | 01-05 | 1.37 | 644 |
| 27-01 | CASSLRTAGYNEQFF | 02-01 | 1.35 | 635 |
| 02-01 | CASSGTRQISGTEAFF | 01-01 | 1.35 | 633 |
| 21-01 | CASSTGLAAQETQYF | 02-05 | 1.32 | 620 |
| 16-01 | CASSQSDLTAKQPQHF | 01-05 | 1.29 | 603 |
| 05-01 | CASSPWTSGGAYNEQFF | 02-01 | 1.2 | 561 |
| 06 | CASRSLLGTRVETEAFF | 01-01 | 1.14 | 535 |
| 05-01 | CASSLWGADGYTF | 01-02 | 1 | 468 |

| 1. **Patient SR/CI-3 at Peak reinfection (Wk 5)** | | | | |
| --- | --- | --- | --- | --- |
| **TRBV** | **CDR3** | **TRBJ** | **Freq. (%)** | **Count** |
| 02-01 | CASSGTRQISGTEAFF | 01-01 | 11.76 | 4708 |
| 10-03 | CAISESTAGSQPQHF | 01-05 | 10.46 | 4187 |
| 27-01 | CASIEQGGDFTDTQYF | 02-03 | 4.84 | 1938 |
| 06-01 | CASSETGQTYEQYF | 02-07 | 4.42 | 1771 |
| 07-09 | CAGNNRDSSTDTQYF | 02-03 | 3.85 | 1541 |
| 19-01 | CASSIRDIGNQPQHF | 01-05 | 3.42 | 1369 |
| 10-03 | CAITDGASGANEQFF | 02-01 | 2.95 | 1182 |
| 07-03 | CASSLVGSGDTQYF | 02-03 | 2.71 | 1086 |
| 04-01 | CASSQEGSDNQPQHF | 01-05 | 2.44 | 975 |
| 11-02 | CASSLILAGGSYNEQFF | 02-01 | 2.33 | 933 |
| 09-01 | CASSGGLINTGELFF | 02-02 | 2.29 | 915 |
| 12 | CASSIAGPPYNEQFF | 02-01 | 1.92 | 770 |
| 20 | CSAREIASSNQPQHF | 01-05 | 1.76 | 703 |
| 06 | CASRSLLGTRVETEAFF | 01-01 | 1.59 | 635 |
| 09-01 | CASSPASSSFYEQYF | 02-07 | 1.58 | 631 |
| 06 | CASSPTGPFYGYTF | 01-02 | 1.37 | 547 |
| 16-01 | CASSQSDLTAKQPQHF | 01-05 | 1.19 | 478 |
| 10-03 | CAISESAVGYNEQFF | 02-01 | 1.19 | 477 |
| 24 | CATSDASLSSYNEQFF | 02-01 | 1.14 | 458 |
| 05-01 | CASSPWTSGGAYNEQFF | 02-01 | 1.11 | 445 |

| 1. **Patient SR/CI-3 at late reinfection (Wk 41)** | | | | |
| --- | --- | --- | --- | --- |
| **TRBV** | **CDR3** | **TRBJ** | **Freq. (%)** | **Count** |
| 20 | CSARPIDSSNQPQHF | 01-05 | 6 | 1737 |
| 10-03 | CAISESTAGSQPQHF | 01-05 | 5.73 | 1658 |
| 02-01 | CASSGTRQISGTEAFF | 01-01 | 4.69 | 1356 |
| 11-02 | CASSLILAGGSYNEQFF | 02-01 | 3.91 | 1130 |
| 02-01 | CASSEIGQLETQYF | 02-05 | 3.85 | 1114 |
| 07-03 | CASSLVGSGDTQYF | 02-03 | 3.36 | 972 |
| 09-01 | CASSSAVIVPGELFF | 02-02 | 3.19 | 923 |
| 27-01 | CASIEQGGDFTDTQYF | 02-03 | 2.53 | 732 |
| 09-01 | CASSGGLINTGELFF | 02-02 | 2.34 | 677 |
| 19-01 | CASSIRDIGNQPQHF | 01-05 | 2.31 | 668 |
| 07-09 | CAGNNRDSSTDTQYF | 02-03 | 2.27 | 656 |
| 12 | CASSIAGPPYNEQFF | 02-01 | 2.03 | 588 |
| 10-03 | CAITDGASGANEQFF | 02-01 | 1.87 | 541 |
| 10-03 | CAISESPQSYEQYF | 02-07 | 1.76 | 508 |
| 02-01 | CASSDPLGQGFSYEQYF | 02-07 | 1.56 | 452 |
| 20 | CSAREIASSNQPQHF | 01-05 | 1.52 | 439 |
| 05-01 | CASSLWGADGYTF | 01-02 | 1.49 | 430 |
| 05-01 | CASRGDTFYEQYF | 02-07 | 1.43 | 415 |
| 09-01 | CASSPASSSFYEQYF | 02-07 | 1.35 | 390 |
| 10-03 | CAISESAVGYNEQFF | 02-01 | 1.14 | 330 |
| 06-01 | CASSDTGTHIYEQYF | 02-07 | 1.11 | 322 |
| 07-03 | CASSSAPGQLETQYF | 02-05 | 1.07 | 309 |
| 04-01 | CASSQEGSDNQPQHF | 01-05 | 1.03 | 299 |
