# Supplementary material for: Selective expansion of high functional avidity memory CD8 T cell clonotypes during hepatitis C virus reinfection and clearance
Source: PLoS Pathog. 2017 Feb 1;13(2):e1006191. doi: 10.1371/journal.ppat.1006191 (PMC5305272; doi:10.1371/journal.ppat.1006191)
Supplement: S8 Table — (DOCX) [file ppat.1006191.s014.docx]

**Table S8. CD8 T cell clones TCR deep sequencing**

| **Clone** | **Amino acid sequence** | **TRBV** | **Sequence frequency (%)** | **Clonotype frequency at peak reinfection (%)** |
| --- | --- | --- | --- | --- |
| **R1** | CASSVDEGNTGELFF | 09-01 | 92.1 | 0.7 |
|  | CSAWTGSATEAFF | 20 | 7.2 | 33.4 |
| **R2** | CASISSNQPQHF | 07-09 | 57.8 | 5.2 |
|  | CASSSGQGNIQYF | 28-01*01 | 35.4 | 3.6 |
| **R3** | CASSLSGGNQETQYF | 27-01*01 | 97.8 | ND |
| **R4** | CASSLSGTGELFF | 12 | 59.2 | 2.5 |
|  | CASSSGQGNIQYF | 28-01*01 | 34.5 | 3.6 |
| **R5** | CATSEPAGLAGGNNEQFF | 24 | 87.4 | < 0.01 |
|  | CASSPGTSDPANYGYTF | 27-01*01 | 12.5 | 8.0 |
| **C1** | CASSLAGTSTEQFF | 14-01*01 | 99.5 | 0.2 |
| **C2** | CASSLEGLVAVETQYF | 05-01*01 | 97.2 | ND |
| **C3** | CASSSLADFYQPQHF | 07-02*01 | 99.9 | ND |
| **C4** | CASSLGTSGGFTDTQYF | 05-06*01 | 99.3 | ND |
| **C5** | CASSYSAGTLDYGYTF | 06-06 | 99.8 | 0.6 |

ND: Not detected
